# Targeting Erbin in B cells for therapy of lung metastasis of colorectal cancer

**DOI:** 10.1038/s41392-021-00501-x

**Published:** 2021-03-12

**Authors:** Tong Shen, Jing-Lin Liu, Chu-Yi Wang, Youlutuziayi Rixiati, Shi Li, Ling-Dong Cai, Yuan-Yuan Zhao, Jian-Ming Li

**Affiliations:** 1 Department of Pathology, Soochow University Medical School, Suzhou, China; 2 Department of Pathology, Sun Yat-sen Memorial Hospital, Sun Yat-sen University, Guangzhou, China

**Keywords:** Gastrointestinal cancer, Cancer microenvironment

## Abstract

The mechanisms and key factors involved in tumor environments for lung metastasis of CRC are still unclear. Here, using clinical samples from lung metastases of CRC patients, we found that intestinal immune network for IgA production was significantly dysregulated in lung metastases of CRC. Single-cell RNA sequencing discovered a subtype of B cells positive for Erbin, one member of the leucine-rich repeat and PDZ domain (LAP) family, was involved in the lung metastases. Erbin deletion in B cells suppressed lung metastasis of CRC in vivo. And, deletion of Erbin in B cells enhanced the killing effects of CD8^+^ T cells on tumor cells. Mechanistically, Erbin knockout attenuated TGFβ-mediated suppression of migration of CXCR5^+^ IgA^+^ cells and STAT6-mediated PD1 expression. Our study uncovered a key role of Erbin in regulating PD1^+^ IgA^+^ B cells in lung metastasis of CRC. Targeting Erbin as well as combined use of neutralizing B cells and antibodies neutralizing PD1 suppresses lung metastasis of CRC in mice, suggesting the potential option for treatment of lung metastasis of CRC.

## Introduction

Colorectal cancer (CRC) is one of the leading causes of cancer death worldwide.^1^ Recently, the most important progress in treatment of CRC may be the treatments of targeting EGFR or targeting PD1/PDL1 based on the discoveries of these biomarkers.^2–5^

The lung is the second most common organ in CRC metastasis. However, the mechanisms for lung metastasis of CRC are still not well-studied.^6^ The lung has its own characteristics in tissue components including airway epithelial cells (AECs), interstitial structural cells, endothelial cells, and various inflammatory cells, such as macrophages, neutrophils, and lymphocytes. Under physiological conditions, both the innate and acquired immune system in the lung microenvironment play the key roles in maintaining tissue homeostasis. In some pathological conditions such as cancer progression, the homeostasis of lung is greatly destroyed.^7^ Nevertheless, the key factors in lung microenvironment involved in lung metastases of CRC are still uncharacterized.

Unlike T lymphocytes and tumor-associated macrophages (TAMs),^8,9^ B cells are now not well-studied in progression and metastasis of cancer. Recent evidences demonstrated that B cells were not the bystanders in the tumor microenvironment, which not only presented antigens to CD4^+^ or CD8^+^ cells, forming antigen-specific immune responses in the tumor microenvironment,^10–12^ but also promoted the further maturation of B cells, the isotype switching of tumor-specific B cells, and the immune response of T cells in tumors by the way of the formation of tumor-associated tertiary lymphoid structure (TLS).^13–15^ B cells infiltrating in the tumor and formation of TLS have key roles in the immune microenvironment in cancer progression.^16–18^ One of the mechanisms suggested that B cells can selectively promote lymph node metastasis by producing pathogenic IgG.^19^

Here, using clinical samples from primary cancer and lung metastases of CRC, we found that intestinal immune network for IgA production was significantly dysregulated in lung metastases of CRC. Single-cell RNA sequencing and functional studies discovered a subtype of B cells positive for Erbin, one member of the leucine-rich repeat and PDZ domain (LAP) family, plays a key role in the lung metastases. Our study uncovered Erbin as a potential target for treatment of lung metastasis of CRC.

## Results

### B cells related genes and pathways are significantly enriched in CRC patients with or without distant metastasis

To find the key gene and pathway involved in metastasis of CRC, we performed transcriptome sequencing to detect differentially expressed genes between tumors and adjacent tissues from CRC cancer patients with or without distant metastasis (Supplementary Table S2a, b).

In CRC cancer patients without distant organ metastasis, we found that the genes related to IgA production or the chemokine signaling pathway were differentially expressed in tumor tissues and adjacent tissues. KEGG enrichment analysis showed that the IgA immune pathway and its B-cell receptor signaling pathway were significantly enriched in adjacent tissues compared with that in tumors (Supplementary Fig. S1b, c). To further study the role of B cells in CRC, expressions of CD19, CD38, and IgA were analyzed in different regions including the adjacent stromal, paracancerous or normal tissue (Norm), invasion margin (IM), and cancer center (CT) of 91 cases of CRC patients without distant metastasis by immunohistochemistry (IHC). CD19^+^ cells, CD38^+^ cells, and IgA^+^ cells showed a gradient decreasing distribution pattern, as these cells were mainly concentrated in the Norm, partly aggregated in IM, and rarely appeared at CT of the tumors (Supplementary Fig. S1a, d). Prognosis analysis indicated that those CRC patients with more CD38^+^ cells in the marginal area of colorectal tumors had better clinical prognosis (Supplementary Fig. S1e).

In CRC cancer patients with distant metastasis, we found that genes related to IgA production were highly expressed in adjacent tissues, and genes related to protein digestion and absorption were highly expressed in tumor tissues (Supplementary Fig. S1g, h). Furthermore, we analyzed expressions of CD19, CD38, and IgA in different regions including Norm, IM, and CT of 117 cases of CRC patients with distant metastasis by IHC. CD19^+^ cells, CD38^+^ cells, and IgA^+^ cells showed similar distribution pattern described in CRC patients without distant metastasis, as these cells were mainly concentrated in the Norm, partly aggregated in IM, and rarely appeared at CT of the tumors (Supplementary Fig. S1f, i). Interestingly, we found that CRC patients with more CD19^+^ cells and IgA^+^ cells in the marginal area of colorectal tumors had better clinical prognosis (Supplementary Fig. S1j).

Briefly, our study showed B cells were dysregulated in CRC patients with or without distant metastasis.

### Immune pathways for IgA production and Erbin^**+**^ B lymphocytes are significantly dysregulated in lung metastasis of CRC

**Fig. 1:**
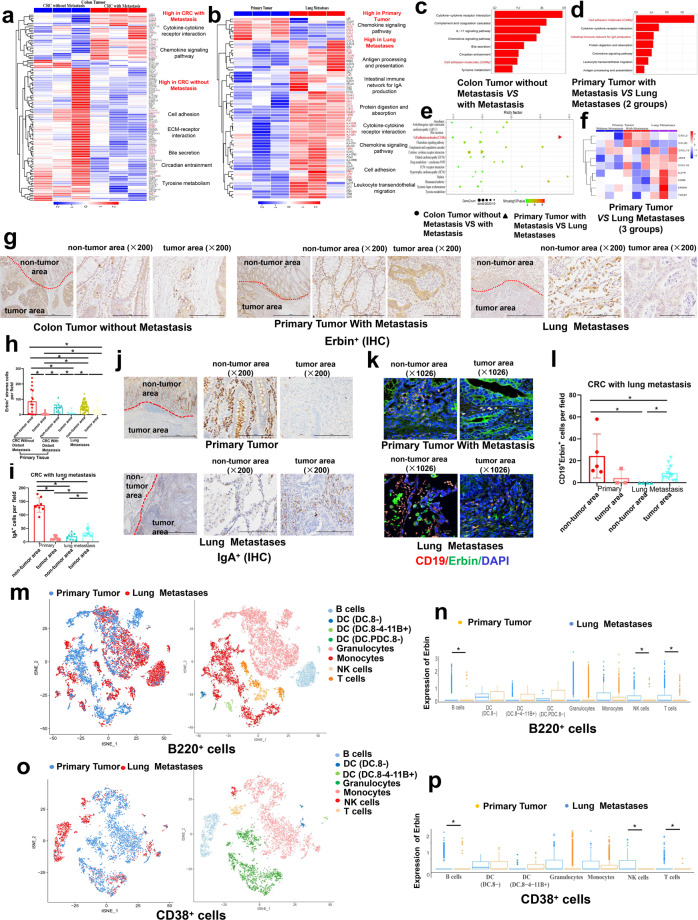
Transcriptome analyses of lung metastases from CRC patients and single-cell RNA sequencing (scRNA-seq) of lung metastases from mouse CRC model. **a**–**f** Heatmap representing differential expressed genes (**a**) and KEGG pathway enrichment analyses in primary tumors from CRC patients with or without distant metastasis (Supplementary Table S1) (**c**), or that in primary tumors and lung metastases from CRC patients (Supplementary Table S1) (**b**, **d**–**f**). **g**–**j** Immunohistochemical staining and semi-quantification of the number of Erbin^+^ stromal cells (**g**, **h**) and IgA^+^ (**i**, **j**) in primary tumors and lung metastases from CRC patients (Supplementary Table S3). The red dotted line indicates the junction area between tumor area and paracancerous area. IgA^+^ and Erbin^+^ cells per field (×200) in CRC patients (**g**, **j**). **k**, **l** Immunofluorescence of Erbin^+^ CD19^+^ cells in primary tumors and lung metastases from CRC patients (Table S3), scale bars, 20 μm (**k**). Semi-quantification of the number of Erbin^+^ CD19^+^ cells by multiple immunofluorescent staining (**l**). **m**t-SNE display and graph-based clustering of B220^+^ cells. **n** Counts of the expression of Erbin in each cluster of B220^+^. **o** t-SNE display and graph-based clustering of CD38^+^ cells. **p** Counts of the expression of Erbin in each cluster of CD38^+^

To find the key genes or pathways involved in distant metastasis of CRC, we compared primary tumors in CRC patients with or without distant metastasis. Notably, immune-related pathways, cell adhesion-related pathway, digestion, and absorption-related pathways, and tyrosine metabolism pathways were greatly enriched by KEGG analysis (Fig. 1a, c) and key genes for each pathway was displayed by Heatmap (Fig. 1a).

To further find the key genes or pathways involved in lung metastasis of CRC, we performed gene expression profiling of primary tumors and lung metastases in CRC patients with lung metastasis. We found that genes interacting with cytokines and their receptors, such as IL-6R, CXCL1, etc, were highly expressed in primary tumors while genes associated with IgA production (IL-21R, TGFβ, etc) and some chemokines (CXCL13) were highly expressed in lung metastases (Fig. 1b). In particular, genes related to the cell adhesion molecules (CAM) pathway (Erbin, SIGLEC1, CDH2, etc) are highly expressed in lung metastases (Fig. 1b, d–f). Among these genes, Erbin, was previously found as Erbb2-interacting protein. KEGG analysis showed that the immune pathway for IgA production were significantly dysregulated in lung metastasis of CRC (Fig. 1b, d). Furthermore, we analyzed expression pattern of IgA in primary tumors and lung metastasis of CRC patients (Supplementary Table S3) by IHC. In the primary tumors, IHC results showed that IgA^+^ cells were significantly enriched in adjacent regions (Fig. 1i, j). Interestingly, in the lung metastasis of CRC, IgA^+^ cells were significantly aggregated in tumor region. In addition, we also analyzed the expression of Erbin in tumor-associated stromal cells by IHC and found Erbin^+^ lymphocytes were significantly accumulated in precancerous regions of lung metastases (Fig. 1g, h). We performed double-staining immunofluorescence for Erbin and CD19 (Fig. 1k) and semi-quantitative analysis (Fig. 1l) in primary tumors and lung metastases from CRC patients. We found similar distribution pattern of Erbin^+^ CD19^+^ cells compared with that of IgA cells in primary tumors and lung metastases from CRC patients. Moreover, we analyzed the expression of Erbin in tumor-associated stromal cells mostly lymphocytes in CRC patients with or without distant metastasis (cohort1 and cohort2, Supplementary Table S2a, b) and found Erbin^+^ lymphocytes were mainly concentrated in the Norm, partly aggregated in IM, and rarely appeared at CT of primary tumors (Supplementary Fig. S2a, b).

To study the components and dynamics of B cells in lung metastasis of CRC, we established a mouse model for lung metastasis of CRC and isolated B220^+^ cells in tumors using BD IMag Mouse B Lymphocyte Enrichment Kit and analyzed these cells using single-cell RNA sequencing (scRNA-seq) involving a single-tube protocol with unique transcript counting through barcoding with unique molecular identifiers (UMIs). Finally, 12588 mouse B220^+^ single cells, 3748 mouse CD38^+^ single cells, and 1588 mouse CD79a^+^ single cells, were successfully obtained for the following analysis. These cells were clustered using Find Clusters in Seurat. About 8 unique clusters labeled with B220^+^, 7 clusters labeled with CD38^+^ were identified by using t-distributed stochastic neighbor embedding (t-SNE) (Fig. 1m, o).

Among 8 clusters of B220^+^ cells, cluster B cells were mainly found in lung metastases (Fig. 1m). Interestingly, Erbin^+^ cells accounted for 22.12% of B220^+^ cells, 22.81% of CD38^+^ cells by Loupe Cell Browser v3.1.0 (Supplementary Fig. S3b). Notably, the expression of Erbin in B cells, T cells, and NK cells clusters from primary tumors and metastases were significantly different (Fig. 1n). We tracked and analyzed the expression and distribution of enriched genes from primary tumors and lung metastases in 8 clusters. The violin diagram showed that these genes were highly expressed in almost 8 clusters except DCs cluster from lung metastases (Supplementary Fig. S3a).

Among 7 clusters of CD38^+^ cells, cluster B cells were mainly found in lung metastases (Fig. 1o). We tracked and analyzed the expression and distribution of enriched genes from primary tumors and lung metastases in 7 clusters. The violin diagram showed that these genes were highly expressed in almost 7 clusters except DCs cluster from lung metastases (Supplementary Fig. S3c). In addition, the expression of Erbin in B cells from primary tumors and metastases were significantly different (Fig. 1p).

In brief, our study showed that Erbin^+^ cells especially Erbin^+^ B lymphocytes are significantly dysregulated in lung metastasis of CRC.

### Erbin deletion leads to aggregation of plasma cells in the lung of mice

**Fig. 2:**
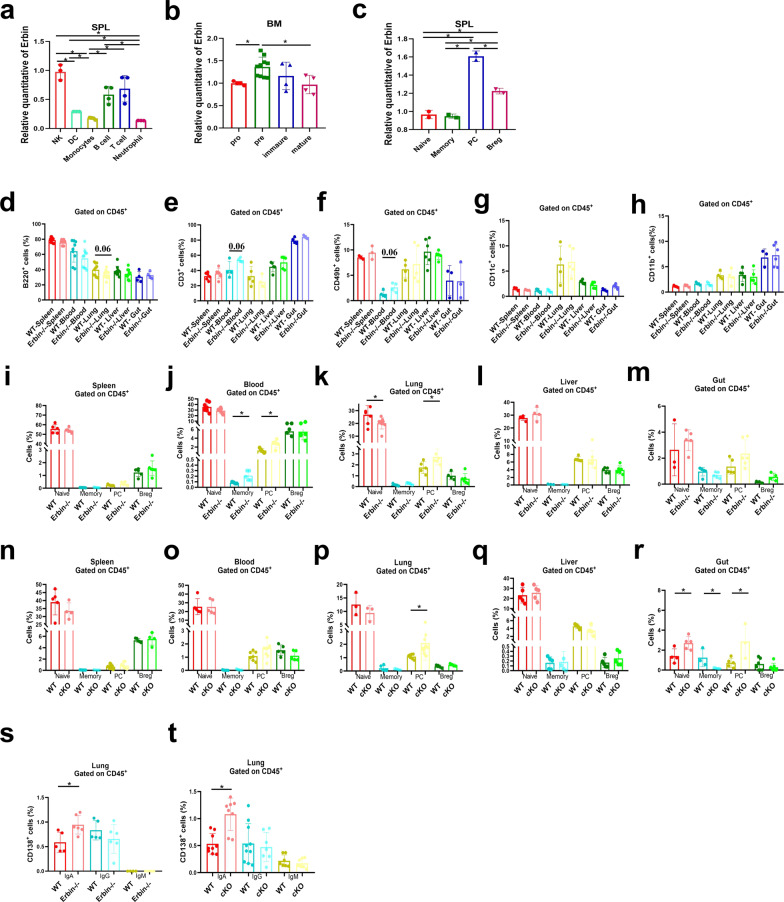
Expression of Erbin in different types of immune cells and the effects of Erbin deletion on immune cells. **a** The mRNA level of Erbin in NK cells (NK), Dendritic cells (DC), Monocytes, B cells, T cells, and Neutrophil cells in splenic leukocytes (SPL). **b** The mRNA level of Erbin in the pro, pre, immature and mature phases of B cells in bone marrow (BM). **c** The mRNA level of Erbin in Naive B, Memory B, Plasma B cells (PC), Breg in splenic leukocytes (SPL). **d**–**h** Proportion of B220^+^ cells, CD3^+^ cells, CD49b^+^ cells, CD11c^+^ cells, and CD11b^+^ cells in leukocytes of spleen, blood, lung, liver, and gut in Erbin full knockout (Erbin^−/−^) mice. **i**–**m** Proportion of Naive B cells, Memory B cells, Plasma cells (PC), and Breg cells of spleen, blood, lung, liver, and gut in Erbin^−/−^ mice. **n**–**r** Proportion of Naive B cells, Memory B cells, Plasma cells (PC), and Breg cells of spleen, blood, lung, liver, and gut in cKO mice. **s**–**t** Proportion of IgA^+^/IgG^+^/IgM^+^ CD138^+^ cells of lung leukocytes in Erbin^−/−^ mice (**s**) and WT vs cKO mice (**t**). *p < 0.05 (Student *t* test)

To understand the role of Erbin in immune cells, we first detected the expression of Erbin in different types of immune cells including natural killer cells (NK), dendritic cells (DC), monocytes, B lymphocytes, T lymphocytes, and neutrophils in spleen of mice. qRT-PCR results showed that Erbin was highly expressed in B cells, T cells, and cells (Fig. 2a). Next, we examined the expression of Erbin at different development stages of B cell. B lymphocytes were isolated from bone marrow and spleen of mice, respectively. Interestingly, we found that Erbin was significantly elevated in pre-B cells stage in bone marrow (Fig. 2b). More importantly, Erbin was highest expressed in plasma cells in spleen of mice (Fig. 2c).

To understand the role of Erbin in B cells development in vivo, we established an Erbin full knockout mouse model based on cas9 targeting strategy (Fig. S2). Immune cells such as NK cells (CD49b^+^), DC (CD11c^+^), monocytes (CD11b^+^), B cells (B220^+^), and T cells (CD3^+^) were detected from the peripheral organs of the spleen, blood, lung, liver and intestine (Fig. 2d–h). Furthermore, when we detected the proportion of different subsets of B cells in each peripheral organ of mice, we found that the proportion of Memory B cells (B220^+^ CD138^−^ IgD^−^) and PC cells (B220^−^ CD138^+^) in blood, and PC cells in the lung of Erbin-deficient mice was significantly elevated than that of control mice (Fig. 2i–m). Because Erbin is highly expressed in other immune cells except for B cells, we established a B-cell conditional Erbin deletion mouse model (Erbin^loxp/loxp^; CD19-cre, abbreviated as cKO) (Fig. S4a, b) to further study the changes of Naive B, Memory B, PC, and Breg cells in spleen, blood, lung, liver, and intestine of mice (Fig. 2n–r). Among these cells, the number of PC cells in lung was dramatically higher (about 3–10 times) in mice with B-cell conditional depletion of Erbin than that in the control mice. In addition, we analyzed the numbers of IgA^+^ CD138^+^, IgG^+^ CD138^+^ and IgM^+^ CD138^+^ cells in lung metastases of Erbin full or B-cell-specific deletion mice and found that the number of IgA^+^ CD138^+^ cells in lung metastases were significantly higher than those in the control group (about three times of that in the control group) (Fig. 2s, t).

In general, Erbin deletion results in aggregation of plasma cells in the lung of both Erbin full knockout and B-cell cKO mice.

### Erbin deletion suppresses lung metastasis of CRC and adoptive cell transfer therapy using B cells isolated from Erbin deletion mice significantly attenuates lung metastasis of CRC in mice

To know the effects of Erbin mediated B cells in lung metastasis of colorectal cancer, we established the lung metastasis mouse models of CRC by tail vein injection of two mouse colorectal cancer cells MC38 and/or CMT93 in Erbin full knockout and B-cell cKO mice.

**Fig. 3:**
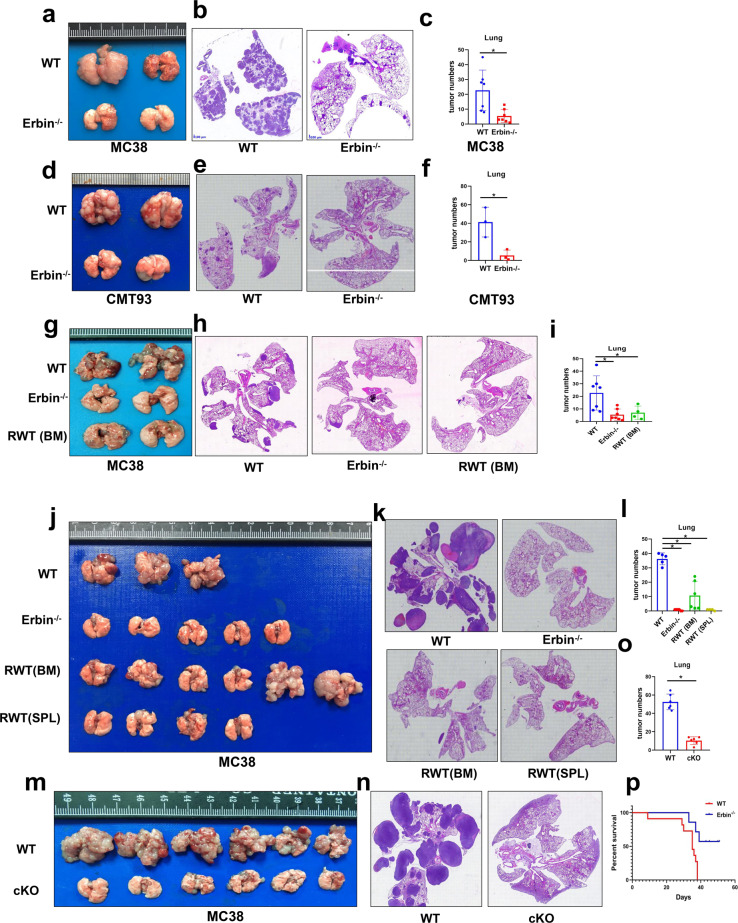
The effects of Erbin deletion or adoptive cell transfer therapy using B cell isolated from Erbin deletion mice in lung metastasis mouse model of CRC. **a**–**c**, Gross lung appearance (**a**), H&E (haematoxylin and eosin) staining (**b**), the numbers of lung metastases (**c**), were determined in Erbin^−/−^ lung metastasis mouse model of CRC one month after administrated with MC38 cells. **d**–**f** Gross lung appearance (**d**), H&E staining (**e**), the numbers of lung metastases (**f**), were determined in Erbin^−/−^ lung metastasis mouse model of CRC one month after administrated with CMT93 cells. **g**–**i** Gross lung appearance (**g**), H&E staining (**h**), the numbers of lung metastases (**i**), in WT control, Erbin^−/−^, RWT (BW) mice one month after administrated with MC38 cells. RWT (BW) mice were WT mice receiving B cells isolated from bone marrow of Erbin^−/−^ mice. **j**–**l** Gross lung appearance (**j**), H&E staining (**k**), the numbers of lung metastases (**l**), in WT control, Erbin^−/−^, RWT (BW), and RWT (SPL) mice 1 month after administrated with MC38 cells. RWT (BW) mice were WT mice receiving B cells from bone marrow of Erbin^−/−^ mice. RWT (SPL) mice were WT mice receiving B cells from spleen of Erbin^−/−^ mice. **m**–**o** Gross lung appearance (**m**), H&E staining (**n**), the numbers of lung metastases (**o**), in WT and cKO mice 1 month after administrated with MC38 cells. **p** Survival curves of WT and Erbin^−/−^ mice within 2 months after administrated with MC38 cells. *p < 0.05 (Student *t* test)

In Erbin full knockout mice, Erbin deletion suppressed lung metastasis of colorectal cancer, and the number of lung metastases was significantly deceased after administration of two mouse colorectal cancer cells MC38 and CMT93 compared with that in wild-type (WT) control mice (Fig. 3a–f). More importantly, adoptive cell transfer therapy using B cell isolated from the bone marrow of Erbin deletion mice significantly attenuated lung metastasis of colorectal cancer in WT control. In vivo, lung metastases assay showed that the phenotype of wild-type control mice transferred by B cells isolated from the bone marrow of Erbin deletion mice was similar to that of the Erbin deletion mice (Fig. 3g–i). Interestingly, adoptive cell transfer therapy using B cell isolated from spleen of Erbin deletion mice also successfully inhibited lung metastasis of colorectal cancer in wild-type control. However, in some mice transferred by B cell isolated from the bone marrow of Erbin deletion mice, lung metastasis was not significantly alleviated compared with that in control mice (Fig. 3j–l). It looked like that the B cell isolated from spleen of Erbin deletion mice inhibited lung metastasis more efficiently than the B cell from the bone marrow of Erbin deletion mice, suggesting that the late stage of B cells in Erbin deletion mice involved in B-cell-mediated tumor immunity of lung metastasis of CRC.

In Erbin B-cell cKO mice, we got similar results to that in Erbin full knockout mice (Fig. 3m–o). Survival analysis indicated Erbin-deficient mice had better survival than WT mice. When most mice died in 40 days after administration of MC38 cells by the tail vein, half of Erbin-deficient mice were still alive even after 2 months after administration of MC38 cells (Fig. 3p).

### The proportion of IgA^+^ CD138^+^ cells was significantly increased in lung metastases of CRC in Erbin-deficient mice

**Fig. 4:**
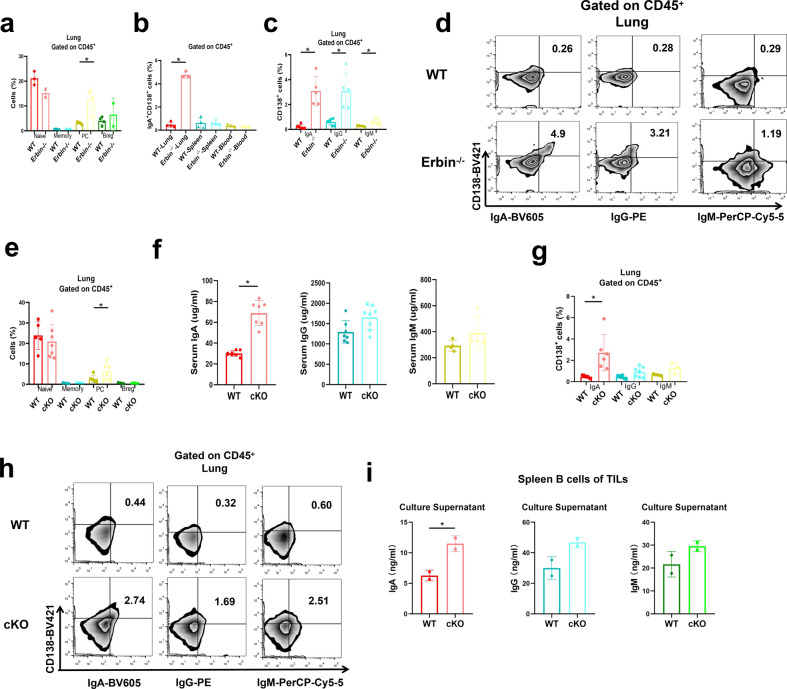
The proportion of IgA+ CD138+ cells in lung metastases of CRC in Erbin-deficient mice. **a** The percentage of Naive B cells, Memory B cells, Plasma cells, and Breg cells in the lung of tumor-bearing WT and Erbin^−/−^ mice. **b** The percentage of IgA^+^ CD138^+^ cells in lung, spleen, and blood leukocytes of tumor-bearing WT and Erbin^−/−^ mice. **c**, **d** The percentage (**c**) and flow cytometry analysis (**d**) of IgA^+^/IgG^+^/IgM^+^ CD138^+^ cells in lung leukocytes of tumor-bearing WT and Erbin^−/−^ mice. **e** The percentage of Naive B cells, Memory B cells, Plasma cells, and Breg cells in the lung of tumor-bearing WT and cKO mice. **f** ELISA analysis of serum IgA/IgG/IgM content in tumor-bearing WT and cKO mice. **g**, **h** The percentage (**g**) and flow cytometry analysis (**h**) of IgA^+^/IgG^+^/IgM^+^ CD138^+^ cells in lung of tumor-bearing WT and cKO mice. **i** ELISA analysis of the content of IgA, IgG, and IgM in the supernatant of spleen B cells from tumor-bearing WT and cKO mice, which were induced into plasma cells in vitro. *p < 0.05 (Student *t* test)

In our above study, we have found that the late-stage B cells in Erbin deletion mice were involved in B-cell-mediated tumor immunity of lung metastasis of CRC, and plasma cells aggregated in the lung of both Erbin full knockout and B-cell cKO mice. Furthermore, we want to know what type of B cells regulated by Erbin was crucially involved in lung metastasis of CRC. We detected different subpopulations of mature B in the lung metastasis of CRC in both Erbin full knockout and B-cell cKO mice and found that the number of plasma cells infiltrating in the lung metastases of CRC was significantly elevated for about 3–10 times in the two Erbin knockout transgenic mice compared with that in the control mice (Fig. 4a, e). However, the number of Breg subpopulations in the lung metastasis of CRC in the two Erbin knockout transgenic mice was not significantly changed compared with that in control mice (Fig. 4a, e).

As we know, plasma cells execute their functions by secreting specific antibodies, thus, we detected the expression levels of IgA, IgG, and IgM immunoglobulin in peripheral blood. Expression levels IgA and IgG in peripheral blood of Erbin B-cell cKO mice were dramatically higher (about three times) than that of the controls (Fig. 4f).

Elevated levels of IgA might be secreted by IgA^+^ plasma cells, next, we analyzed the proportion of surface-expressing IgA^+^ CD138^+^ cells in lung metastases, spleen, and blood of mice. The numbers of IgA^+^ CD138^+^ cells in the spleen and peripheral blood were not significantly changed in two groups (Fig. 4b). We further analyzed the proportions of the three immunoglobulin-positive cells in total lymphocytes in lung metastases of Erbin^−/−^ or cKO mice (Fig. 4c, d, g, h). In lung metastases, the number of IgA^+^ CD138^+^ cells, IgG^+^ CD138^+^ cells, and IgM^+^ CD138^+^ cells in Erbin^−/−^ mice were significantly higher than those in the control mice (Fig. 4c, d). Among these cells, the number of IgA^+^ CD138^+^ cells in Erbin^−/−^ mice was 20 times higher, while the number of IgG^+^ CD138^+^ cells was 10 times higher than those in control mice. In cKO mice, the number of IgA^+^ CD138^+^ cells, IgG^+^ CD138^+^ cells, and IgM^+^ CD138^+^ cells in lung metastases were higher than those in control mice (Fig. 4g, h). Among these cells, the number of IgA^+^ CD138^+^ cells were about eight times higher than that in the control mice. When we isolated B cells from cKO and control mice for 1-week in vitro culture, we found that the concentration of IgA but not IgG and IgM in the supernatant of cell culture isolated from cKO mice were significantly higher than that in the control group (Fig. 4i).

### IgA^+^ CD138^+^ cells aggregated in lung metastases of CRC in Erbin-deficient mice are most characterized as low expression of PD1

**Fig. 5:**
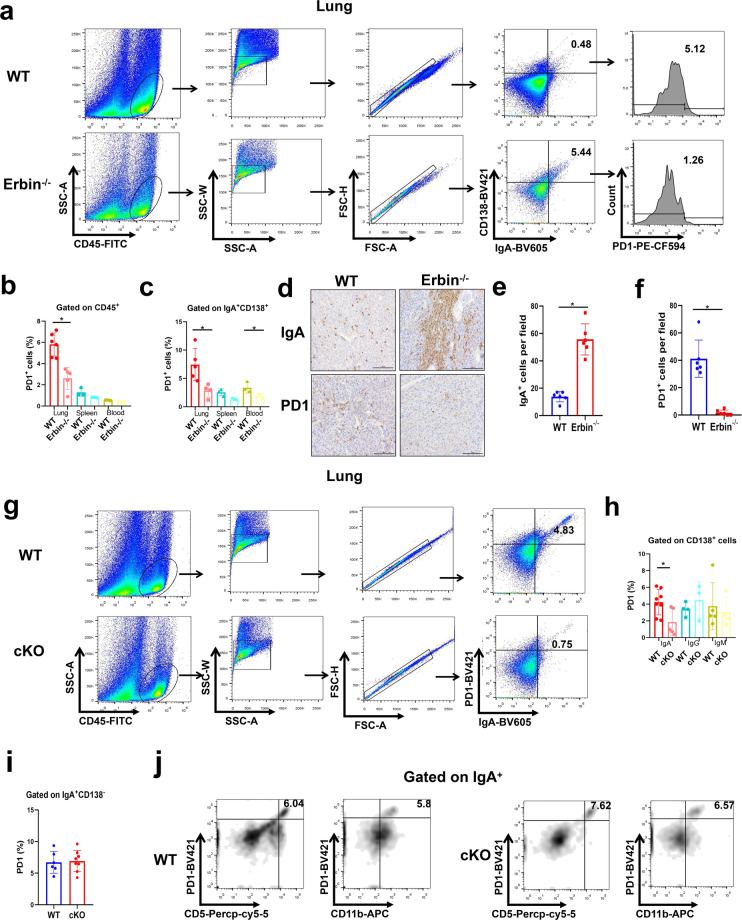
PD1 expression in IgA+ CD138+ cells in lung metastases of CRC in Erbin-deficient mice. **a** The flow cytometry analysis of PD1^+^ cells gated on IgA^+^ CD138^+^ cells in lung of tumor-bearing WT and Erbin^−/−^ mice. **b** The percentage of PD1^+^ cells in leukocytes of lung, spleen, and blood from tumor-bearing WT and Erbin^−/−^ mice. **c** The percentage of PD1^+^ cells gated on IgA^+^ CD138^+^ cells in lung, spleen, and blood of tumor-bearing WT and Erbin^−/−^ mice. **d**–**f** Immunohistochemical staining of IgA and PD1 and semi-quantification of the number of IgA^+^ (**e**) and PD1^+^ cells (**f**) in lung metastases from tumor-bearing WT and Erbin^−/−^ mice. Scale bar, 100 μm. **g**–**j** The flow cytometry analysis of PD1^+^ IgA^+^ cells gated (**g**), the percentage of PD1^+^ cells gated on IgA^+^/IgG^+^/IgM^+^ CD138^+^ cells (**h**), the percentage of PD1^+^ cells gated on IgA^+^ CD138^−^ cells (**i**), the flow cytometry analysis of CD5^+^/CD11b^+^ PD1^+^ cells gated on IgA^+^ cells (**j**), in lung leukocytes of tumor-bearing WT and cKO mice. *p < 0.05 (Student *t* test)

To study the immune characteristics of IgA^+^ CD138^+^ cell subsets in lung metastases, we analyzed the number of PD1^+^ cells in the lung metastases, spleen, and blood of Erbin^−/−^ mice by flow cytometry, and found that the number of PD1^+^ lymphocytes cells in the above organ especially lung was markedly lower than that of the control mice (Fig. 5a, b). Further, we studied the ratio of PD1^+^ in IgA^+^ CD138^+^ cells in the above three organs. Flow cytometric analysis showed that the number of PD1^+^ cells in IgA^+^ CD138^+^ cells of the three organs was significantly lower in Erbin^−/−^ mice than that in the controls (Fig. 5c). Especially, the number of PD1^+^ IgA^+^ CD138^+^ cells in the lung of control mice was about 4 times as more as that in Erbin^−/−^ mice (Fig. 5a, c). IHC results showed that the number of IgA^+^ cells in the lung metastases of Erbin^−/−^ mice was significantly more than that of the control group, while the number of PD1^+^ cells was significantly less than that of the control group (Fig. 5d–f).

In cKO mice, flow cytometric analysis indicated that the number of PD1^+^ in IgA^+^ cells of lung metastases tended to decrease to 1/5 compared with that in control mice (Fig. 5g). Furthermore, when we analyzed the number of PD1^+^ cells in the populations of IgA^+^ CD138^+^, IgG^+^ CD138^+^, IgM^+^ CD138^+^ cells, we found that the number of PD1^+^ IgA^+^ CD138^+^ was significantly decreased in lung metastases of cKO mice compared with that of the control mice (Fig. 5h). The number of PD1^+^ IgA^+^ CD138^+^ cells of lung metastases in Erbin B-cell cKO mice was only equivalent to half of that in control mice (Fig. 5h). The number of PD1^+^ IgG^+^ CD138^+^, PD1^+^ IgM^+^ CD138^+^ was not changed significantly in cKO mice compared with that of the control mice (Fig. 5h). Additionally, the number of PD1^+^ IgA^+^ CD138^−^ was not changed significantly in the lung metastases of the cKO mice compared to that of the control mice (Fig. 5i). In these IgA^+^ cells of the lung metastases, we found that the proportions of PD1^+^ CD5^+^ and PD1^+^ CD11b^+^ cells from cKO mice were not significant changed compared with that in control mice (Fig. 5j). In short, our study showed that the subset of IgA^+^ CD138^+^ cells functioned in lung metastasis of CRC and Erbin deletion led to aggregation of IgA^+^ CD138^+^ with low expression of PD1.

### Deletion of Erbin in B cells enhances the killing effects of CD8^+^ T cells on tumor cells

**Fig. 6:**
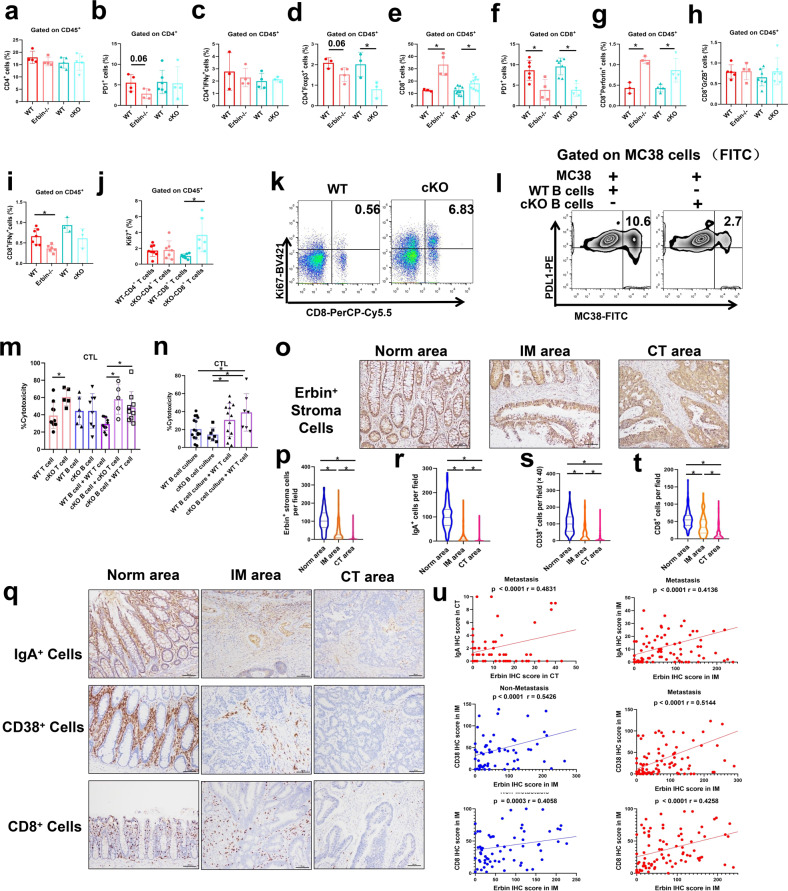
The numbers and functions of CD4+ and CD8+ T cells in Erbin-deficient mice. **a**–**h** The percentages of CD4^+^ cells (**a**), CD4^+^ IFNγ^+^ cells (**c**), CD4^+^ Foxp3^+^ cells (**d**), CD8^+^ cells (**e**), CD8^+^ perforin^+^ cells (**g**), CD8^+^ GrzB^+^ cells (**h**) and **i** CD8^+^ IFNγ^+^ cells gated on lung leukocytes in tumor-bearing WT, Erbin^−/−^, WT, and cKO mice. The percentages of PD1^+^ cells gated on CD4^+^ cells (**b**) and CD8^+^ cells (**f**) in tumor-bearing WT, Erbin^−/−^, WT, and cKO mice. **j** The percentages of CD4^+^ Ki67^+^ and CD8^+^ Ki67^+^ cells gated on lung leukocytes in tumor-bearing WT, and cKO mice. **k** The flow cytometry analysis of CD8^+^ Ki67^+^ cells gated on lung leukocytes in tumor-bearing WT, and cKO mice. **l** The flow cytometry analysis of PDL1 expression in MC38-FITC cells, which were co-cultured with spleen B cells isolated from WT and cKO mice for 3 days, respectively. **m** The cytotoxic activity of tumor cells stimulated by CD8^+^ T cells from WT or cKO mice, B cells from WT or cKO mice, B cells from WT mice co-cultured with CD8^+^ T cells from WT mice, B cells from cKO mice co-cultured with CD8^+^ T cells from cKO mice, and B cells from cKO mice co-cultured with CD8^+^ T cells from WT mice, respectively. **n** The cytotoxic activity of tumor cells stimulated by the supernatant of B cells from WT or cKO mice, the supernatant of B cells from WT mice co-cultured with CD8^+^ T cells from WT mice, and the supernatant of B cells from cKO mice co-cultured with CD8^+^ T cells from WT mice. **o**, **p** Immunohistochemical staining (**o**) and semi-quantification of the number of Erbin^+^ stroma cells in Norm area, IM area, and CT area (**p**) from CRC patients (cohort1 and cohort2). Erbin^+^ stroma cells per field (×400). **q**–**t** Immunohistochemical staining (**q**) and semi-quantification of the number of IgA^+^, CD38^+^, and CD8^+^ cells in Norm area, IM area, and CT area (**r**–**t)** from CRC patients (cohort1 and cohort2). **u** Pearson correlation between IgA and Erbin, CD38 and Erbin, CD8 and Erbin in expression in CRC patients (cohort1 and cohort2). *p < 0.05 (Student *t* test)

We investigated whether Erbin deletion, especially deletion in B cells, will exert its effects for killing tumor cells by T cells in Erbin^−/−^ and cKO mouse models. The results showed that the numbers of CD4^+^ T cells (Fig. 6a), CD4^+^ PD1^+^ cells (Fig. 6b), and CD4^+^ IFNγ^+^ cells (Fig. 6c) in the lung metastasis of Erbin^−/−^ mice and cKO mice were not significantly different from that of the control mice. Interestingly, the numbers of Treg (CD4^+^ Foxp3^+^) cells in lung metastases from the two types of Erbin knockout mice were lower in that from control mice (Fig. 6d).

Importantly, the numbers of CD8^+^ T cells (Fig. 6e) were significantly higher while PD1^+^ CD8^+^ T cells (Fig. 6f) were significantly lower in the lung metastases from the two Erbin-deficient mice than that from control mice. Furthermore, we investigated the changes of functional CD8^+^ T cells, which secreted the killer molecules Perforin, Granzyme B (GrzB), and Interferon-γ (IFNγ) in these animal models. Our results demonstrated that the number of CD8^+^ T cells secreting Perforin was significantly higher in the lung metastases of Erbin-deficient mice than that of control mice (Fig. 6g). The number of CD8^+^ T cells secreting GrzB was not changed significantly in lung metastases of Erbin-deficient mice and control mice (Fig. 6h). The number of CD8^+^ T cells secreting IFNγ was significantly lower in the lung metastasis of Erbin^−/−^ mice than that of control mice, and the number of CD8^+^ T secreting IFNγ in lung metastasis of B cells specific Erbin deletion mice tends to decrease compared with that of control mice although the difference was not statistically significant (Fig. 6i). These results suggested that deletion of Erbin or deletion of Erbin in B cells exerted its effects for killing tumor cells by the help of CD8^+^ T cells secreting Perforin.

Further, we determined the main effects of B-cell-specific deletion of Erbin on the proliferation of CD4^+^ T cells and CD8^+^ T cells in lung metastases of animal models. We found that the proliferation of CD4^+^/CD8^+^ T cells was increased significantly in lung metastasis of B cells specific Erbin deletion mice compared with that of control mice (Fig. 6j, k), further supporting that Erbin-deficient B cells acted its effects mainly by affecting CD8^+^ T cells.

Can Erbin-deficient B cells directly affect the tumor cells in the metastatic lesion? To answer this question, we co-cultured Erbin-deficient B cells with MC38 cells, a kind of mouse colon cancer cell line. The results showed that Erbin-deficient B cells dramatically inhibited the expression of PDL1 in MC38 cells (Fig. 6l).

Our above results showed that Erbin-deficient B cells mainly promoted the proliferation of CD8^+^ T cells and inhibited the expression of PDL1 in colorectal cancer cells. Can Erbin-deficient B cells kill tumor cells by modulating CD8^+^ T cell function? Further, we isolated CD8^+^ T cells and B cells in lung metastasis mice models from cKO and control mice, and co-cultured these cells with MC38 cells, respectively. CTL assay showed that only B cells, neither wild-type B cells nor Erbin-deficient B cells showed no difference in tumor cell killing. Interestingly, CD8^+^ T cells isolated from B-cell-specific Erbin-deficient mice showed more potent in killing tumor cells than CD8^+^ T cells isolated from wild-type control mice. Due to CD8^+^ T cells from B-cell Erbin-deficient mice were not genetically modified, and thus the effect of CD8^+^ T cells isolated from B-cell Erbin-deficient mice to kill tumor cells was activated in vivo by other cell subpopulations, particularly Erbin-deficient B cells. When we co-cultured B cells of cKO mice with CD8^+^ T cells wild-type mouse and tumor cells, the effects of killing tumor cells were close to the effect of co-culturing B cells and CD8^+^ T cells both from cKO mouse, with tumor cells (Fig. 6m), further supporting the key regulatory effects of Erbin-deficient B cells in the function of CD8^+^ T cells in killing tumor cells.

In our study, Erbin-regulating B cells were mainly a type of plasma cells, and the main function of plasma cells was to secrete antibodies. We further examined whether the previously identified Erbin-deficient B-cell-mediated CD8^+^ T cells maximizing killing tumors was related to the antibody secreted by the B cells. We isolated B cells from cKO mice and their control mice, induced them into plasma cells in vitro, and then collected cell supernatants for further study. CTL assays showed that supernatants from B cells of cKO mice increased the effects of CD8^+^ T cells from wild-type mouse on killing tumor cells than supernatants from B cells of control mice although there was no significantly difference (Fig. 6n), further supporting the key regulatory effects of Erbin-deficient B cells in function of CD8^+^ T cells in killing tumor cells.

Expressions of Erbin, IgA, CD38, and CD8 were analyzed in different regions including Norm, IM, and CT of 208 cases (cohort1 and cohort2) of CRC patients by IHC. The results showed that Erbin^+^ lymphocytes (Fig. 6o, p) as well as IgA^+^, CD38^+^, and CD8^+^ cells (Fig. 6q-t), were mainly concentrated in the Norm, partly aggregated in IM, and rarely appeared at CT of the tumors. Correlation analysis showed that the number of IgA^+^ cells was positively correlated with the number of Erbin^+^ lymphocytes in the regions of CT and IM of tumors among CRC patients with distant metastasis. In IM region of tumors, the number of Erbin^+^ lymphocytes in IM region of tumors was positively correlated with the numbers of CD8^+^ and CD38^+^ cells (Fig. 6u).

### Erbin knockout attenuates TGFβ mediated suppression of migration of IgA^+^ CXCR5^+^ cells and inhibits STAT6-mediated PD1 expression

**Fig. 7:**
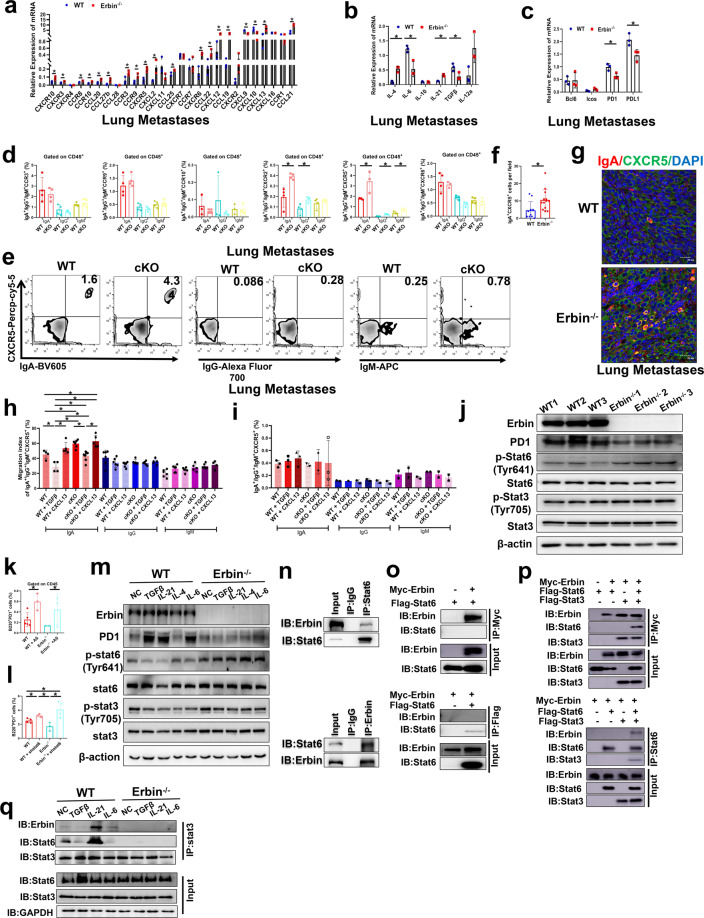
The effects of Erbin on TGFβ mediated suppression of migration of IgA+ CXCR5+ cells and STAT6-mediated PD1 expression in mouse model of lung metastasis of CRC. **a**–**c** qRT-PCR analysis of chemokines and chemokine receptors (**a**), cytokines associated with B-cell differentiation (**b**), co-stimulators or checkpoint inhibitors (**c**) in B cells isolated from lung metastases of WT and Erbin^−/−^ mice (At least 3 mice per group, and data represent mean of 3 independent experiments). **d** Flow cytometry analysis of chemokine receptors closely related to IgA development or function expressed in IgA^+^/IgG^+^/IgM^+^ cells gated on leukocytes in lung metastases of WT and cKO mice. **e** The flow cytometry analysis of IgA^+^/IgG^+^/IgM^+^ CXCR5^+^ cells gated on leukocytes in lung metastases of WT and cKO mice. **f** Semi-quantification of the number of IgA^+^ CXCR5^+^ cells of lung metastases of WT and Erbin^−/−^ mice (*n* = 5) by immunofluorescent staining. **g** Immunofluorescence of IgA^+^ CXCR5^+^ cells of lung metastases of WT and Erbin^−/−^ mice), scale bars, 20 μm. **h**–**i** Effects of TGFβ, CXCL13 on migration of CXCR5^+^ IgA^+^/IgG^+^/IgM^+^ B cells, and B cells were isolated from spleen B cells of tumor-bearing WT and cKO mice. **j** Protein expressions of Erbin, PD1, total or Tyr641 phosphorylated STAT6, total or Tyr705 phosphorylated STAT3 in activated B cells isolated from spleen of WT and Erbin^−/−^ mice by western blotting. **k**, **l** Changes of the percentage of B220^+^ PD1^+^ cells in B cells isolated from spleen of untreated WT and Erbin^−/−^ mice after treated by STAT6 inhibitor AS1517499 (**k**) stimulation or in 293T cells treated by shRNA lentivirus targeting STAT6 (**l**). **m** Protein expressions of Erbin, PD1, total or Tyr641 phosphorylated STAT6, total or Tyr705 phosphorylated STAT3 in B cells isolated from spleen of WT and Erbin^−/−^ mice when stimulated with TGFβ, IL-21, IL-4, and IL-6 by western blotting. **n** Interaction between Erbin and STAT6 in spleen leukocytes from mice by Co-IP assay. **o** Interaction between Erbin and STAT6 by Co-IP assay in 293T cells, in which Myc-Erbin and Flag-STAT6 were overexpressed simultaneously. **p** Interaction between Erbin and STAT6 by Co-IP assay in 293T cells, in which Myc-Erbin, Flag-STAT3, and Flag-STAT6 were overexpressed simultaneously. **q** The effects of TGFβ, IL-21, and IL-6 on interaction between Erbin and STAT6 by Co-IP assay. *p < 0.05 (Student *t* test)

We further investigated the mechanisms by which more IgA^+^ cells aggregated in the lung metastases of Erbin B-cell-specific knockout mice, for example, whether Erbin deletion in B cells will help B cells to differentiate into IgA^+^ B cells or promote migration and chemotaxis of IgA^+^ B cells to lung metastases. In addition, which factors in the tumor microenvironment of lung metastases were involved in the regulation of these above key process? To answer these questions, we first extracted tissue RNA and analyzed expressions of key factors including chemokines and chemokine receptors, pro-inflammatory cytokines involved in B cells development and activation, and immune checkpoints in the lung metastasis of CRC in Erbin-deficient and control mice (Fig. 7a–c). We selected these highly differentially expressed chemokine receptors in Erbin knockout mice, which may be functionally associated with B cells for further study. Then, we used flow cytometry to label and analyze the number of CCR3^+^, CCR9^+^, CCR10^+^, CXCR2^+^, CXCR5^+^, and CXCR6^+^ cells expressed different isotype antibodies including IgA, IgG, and IgM in the lung metastases of cKO and control tumor-bearing mice. We found that the numbers of IgA^+^ CXCR2^+^, IgG^+^ CXCR2^+^, IgA^+^ CXCR5^+^, IgG^+^ CXCR5^+^, and IgM^+^ CXCR5^+^ cells were significantly elevated in cKO (Fig. 7d). Notably, in lung metastases of cKO and control mice, the ratios of IgG^+^ CXCR5^+^ and IgM^+^ CXCR5^+^ in CD45^+^ cells were very low, and IgA^+^ CXCR5^+^ cells population may be the majority of CXCR5^+^ cells. IgA^+^ CXCR5^+^ cells were significantly increased in cKO mice (Fig. 7e). In addition, immunofluorescence staining assay showed that the number of IgA^+^ CXCR5^+^ cells in the lung metastasis of Erbin-deficient mice was significantly higher than that of the control mice (about three times of controls) (Fig. 7f, g). These results suggested that the deletion of Erbin in B cells lead to aggregation of IgA^+^ cells in lung metastases of CRC mouse models.

As we know, the ligand of CXCR5 is CXCL13, which is key for chemotactic migration of B lymphocytes. In addition, TGFβ, as a crucial factor in the tumor microenvironment, plays a very important role in tumor progression and metastasis. Besides, we found that the ligand CXCL13 of CXCR5 in the lung metastases of Erbin-deficient mice was significantly increased compared with that of control mice, while TGFβ was significantly decreased in the lung metastases of Erbin-deficient mice.

We further used cell migration experiments to clarify the regulatory effect of Erbin on CXCL13 or TGFβ primed migration of IgA^+^ CXCR5^+^ cells to mimic their roles in the microenvironment of lung metastases. Erbin-deficient and control B cells were treated with CXCL13 and TGFβ, respectively. The results showed that CXCL13 could significantly increase migration of IgA^+^ CXCR5^+^ cells from WT mice, and Erbin deletion promoted CXCL13 to mediate increasing migration of IgA^+^ CXCR5^+^ cells (although the difference is not statistically significant). Amazingly, TGFβ can significantly reduce the migration rate of IgA^+^ CXCR5^+^ cells from WT mice, and the effects of the same dose of TGFβ on migration of IgA^+^ CXCR5^+^ cells (isolated from cKO) were significantly attenuated after Erbin deletion. The cell migration rate of IgA^+^ CXCR5^+^ after Erbin deletion (isolated from cKO) was about 2-fold higher than that from WT mice. At the same time, we found that the migration abilities of IgG^+^ CXCR5^+^ cells and IgM^+^ CXCR5^+^ cells were not affected by treatments of CXCL13 or TGFβ (Fig. 7h). Moreover, we found that treatments of CXCL13 or TGFβ did not significantly affect the differentiation of B cells into CXCR5^+^ cells expressed different isotypes of antibodies including IgA, IgG, and IgM (Fig. 7i).

In short, our results indicated that Erbin knockout abolishes TGFβ-mediated suppression of migration of IgA^+^ CXCR5^+^ cells and strengthens CXCL13-mediated promotion of migration of IgA^+^ CXCR5^+^ cells, which are responsible for increased IgA^+^ CXCR5^+^ cells in the foci of lung metastasis after Erbin deletion.

When we analyzed these differentially expressed cytokines in the lung metastases of mice, we found that cytokines that regulated activations of STAT3 and STAT6 were significantly enriched. Furthermore, we isolated splenic B cells in Erbin-deficient mice and control mice at background levels. In vitro activation experiments showed that the expression of PD1 in B cells from Erbin-deficient mice was significantly lower, while the expression of p-STAT6 (Try641) in B cells from Erbin-deficient mice was higher than that from control mice (Fig. 7j). Moreover, STAT6 inhibitor AS1517499 or STAT6 specific shRNA significantly restored the number of B220^+^ PD1^+^ from Erbin-deficient mice (Fig. 7k, l), supporting that the decreased expression of PD1 caused by Erbin deletion was closely related to the STAT6 pathway.

To further uncover the cellular and molecular mechanisms by which Erbin regulates PD1 via STAT6 pathway, we analyzed the differentially expressed cytokines in lung metastases and found TGFβ, IL-21, IL-4, and IL-6 were significantly changed in lung metastases from Erbin-deficient mice compared with that from control mice.

In the lung metastasis model of mice, the contents of TGFβ and IL-6 in the lung metastases of WT mice was significantly higher than that of Erbin deletion mice (Fig. 7b). In WT mouse B cells, TGFβ or IL-6 can significantly increase PD1 expression; but in Erbin deletion mouse B cells, Erbin deficiency abolished the increasing effects of TGFβ or IL-6 on PD1 expression. Mechanistically, in WT mouse B cells, TGFβ or IL-6 can significantly inhibit the levels of p-STAT6 (Try641) but in Erbin deletion mouse B cells, Erbin deficiency abolished the inhibitory effects of TGFβ or IL-6 on the level of p-STAT6 (Try641) (Fig. 7m).

In the lung metastasis model of mice, the contents of IL-4 and IL-21 in the lung metastases of WT mice were significantly lower than those of Erbin-deficient mice (Fig. 7b). In WT mouse B cells, IL-21 can significantly increase PD1 expression; but in Erbin deletion mouse B cells, Erbin deficiency attenuated the increasing effects of IL-21 on PD1 expression. Either in B cells from WT mice or in B cells from Erbin deletion mice, IL-4 cannot significantly increase PD1 expression. In WT mouse B cells, IL-4 even slightly inhibited PD1 expression, suggesting that IL-4 did not play a major role in the regulation of PD1 expression. Mechanistically, in WT mouse B cells, IL-21 can significantly inhibit the levels of p-STAT6 (Try641). Erbin deficiency attenuated the inhibitory effects of IL-21 on the level of p-STAT6 (Try641) (Fig. 7m).

We guessed that there would be a direct interaction between Erbin and STAT6. In the endogenous cell system, we used STAT6 and Erbin antibodies for co-immunoprecipitation assay, respectively, and found that there might be a direct interaction between STAT6 and Erbin (Fig. 7n). However, when we overexpressed Erbin and STAT6 at the same time, we cannot reproduce this finding in exogenous cell system, suggesting that the interaction between Erbin and STAT6 might be mediated by the direct interaction between Erbin and other key protein (Fig. 7o). Interestingly, we found that Erbin could interact with STAT6 when STAT3 was overexpressed simultaneously (Fig. 7p). Afterward, to determine which cytokine affected the interaction between Erbin-STAT3-STAT6, we added TGFβ, IL-21, and IL-6 to the splenic lymphocytes from WT and Erbin^−/−^ mice, respectively, by observing the amount of STAT6 and Erbin protein bounded to STAT3. The results showed that IL-21 could enhance the interplay among Erbin-STAT3-STAT6 in splenic lymphocytes and Erbin deficiency significantly abolished interaction between STAT3-STAT6 (Fig. 7q).

In summary, the above results indicated that Erbin knockout promoted migration of IgA^+^ CXCR5^+^ cells and inhibited PD1 expression of IgA^+^ cells via chemokine especially IL-21 induced STAT6 activation.

### Targeting Erbin as well as combined use of neutralizing B cells and antibodies neutralizing PD1 suppress lung metastasis of CRC in mice

**Fig. 8:**
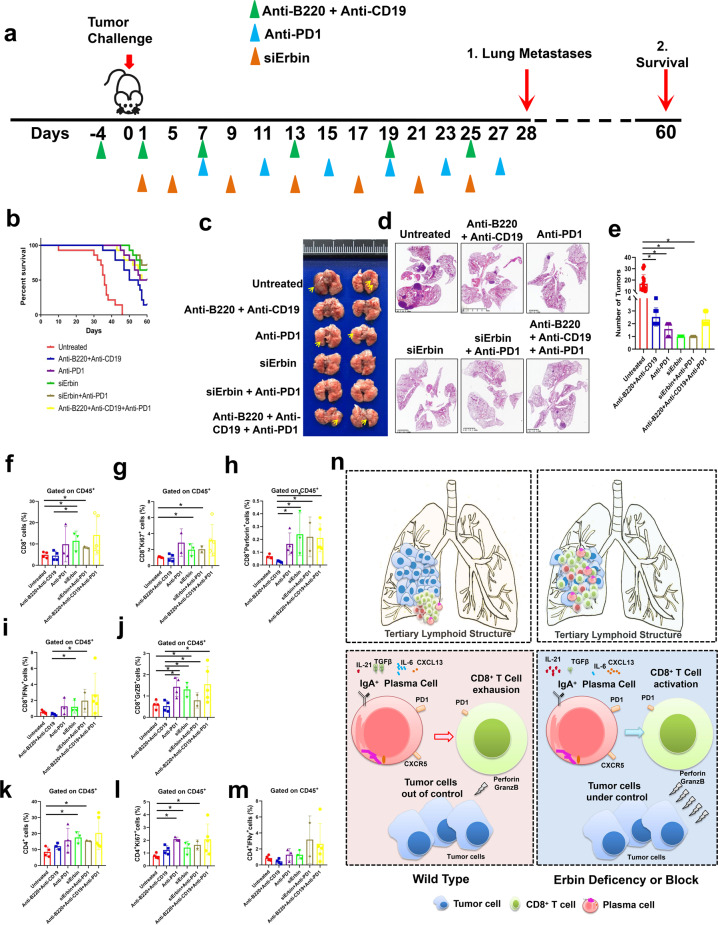
Experimental treatments by targeting Erbin as well as combined use of neutralizing B cells and antibodies neutralizing PD1 in mouse model of lung metastasis of CRC. **a** Schematic diagram showed MC38-bearing C57BL/10 recipient mice received antibody as described in section of “Materials and methods”. **b** The survival curves of tumor-bearing mice treated with targeting Erbin as well as combined use of neutralizing B cells and antibodies neutralizing PD1 (untreated, anti-B220 + anti-CD19, anti-PD1, siErbin, siErbin + anti-PD1, and anti-B220 + anti-CD19 + anti-PD1; *n* = 5 in each group) in mouse model of lung metastasis of CRC. **c**–**e** Gross lung appearance (**c**), H&E staining (**d**), the numbers of lung metastases (**e**), in mice with different treatment one month after administrated with MC38 cells. **f**–**m** The percentages of CD8^+^ T cells, CD8^+^ Ki67^+^ cells, CD8^+^ Perforin^+^ cells, CD8^+^ IFNγ^+^ cells, CD8^+^ GrzB^+^ cells, CD4^+^ T cells, CD4^+^ Ki67^+^ cells, and CD4^+^ IFNγ^+^ cells in lung metastases of mice with different treatment. **n** Schematic diagram for the role of Erbin in lung metastasis of CRC. *p < 0.05 (Student *t* test)

To test the effects of targeting Erbin in mouse model of lung metastasis of CRC, we used in vivo siRNA system targeting Erbin (Fig. S5) and/or combination with multiple neutralizing antibodies including anti-CD19 and anti-B220 antibodies, neutralizing PD1 antibodies, and siRNAs targeting the Erbin gene to analyze their simple or combined effects on lung metastasis of CRC (Fig. 8a). We found that the two-month survival rate of mice in five-treatment groups including inhibiting B-cell function (anti-B220, anti-CD19), neutralizing PD1, targeting Erbin alone (siErbin), targeting Erbin in combination with neutralizing PD1, and simultaneously inhibiting B-cell function and neutralizing PD1, were significantly higher than that in the untreated mice. Notably, we observed that mice targeting Erbin alone had very good prognosis. The 60-day survival rate of about 70% of mice targeting Erbin alone or combination with targeting Erbin and neutralizing PD1 were still alive after 60 days in mouse model of lung metastasis of CRC (Fig. 8b). Consistent with the above results, morphological results showed that mice in five experimental treatment groups had fewer lung metastases than that in the untreated group. Amazingly, only a very small metastasis formed in mice of groups targeting Erbin alone or combination with targeting Erbin and neutralizing PD1, under the microscope (Fig. 8c–e).

Furthermore, we analyzed the number of T cells and their secretion of killer molecules in the lung metastases of the above 6 groups of mice by flow cytometry. Our results showed that the CD4^+^ cells and CD8^+^ T cells increased significantly in mice of groups targeting Erbin alone or combination with targeting Erbin and neutralizing PD1 (Fig. 8f, g, k, l). In addition, in the PD1 neutralization group, the targeting Erbin group (siErbin), the targeting Erbin and PD1 group, and targeting the B cells and PD1 group, CD8^+^ T cells secreting GrzB and Perforin were greatly increased (Fig. 8h–j, m).

## Discussion

Besides liver metastasis, lung metastasis is also very common in CRC patients especially in patients with rectal cancer. However, the biology of lung metastasis of CRC is still poorly understood compared with that of liver metastasis of CRC. More importantly, the key factors in tumor microenvironments of lung metastases of CRC are still uncharacterized.

Here, we found that immune pathway for IgA production and B lymphocytes are significantly dysregulated in lung metastasis of CRC. By far, the physiological function of IgA is very important for homeostasis of gut or lung. Patients with IgA deficiency are usually accompanied by immunodeficiency syndrome.^20,21^ Notably, IgA is the most abundant antibody subtype secreted by the mucosa in gut or lung, which creates a critical coexistence place for interaction between the host and commensal bacteria.^22,23^ In cancer, as early as in 1987, some studies discovered sIgA was significantly elevated in patients with lung cancer, head and neck cancer, and nasopharyngeal carcinoma.^24^ In colon cancer, the recruitment of IgA-secreting plasma cells to tumor-associated mucosa was found in patients.^25,26^ In addition, IgA was significantly increased in the serum of patients with hepatic carcinoma metastasis.^27^ Here, using clinical samples including primary cancer and lung metastases from CRC patients, we found that immune pathways for IgA production were significantly dysregulated in lung metastasis of CRC. Interestingly, we found that more IgA^+^ cells were accumulated in the paracancerous regions of primary cancer from CRC patients with or without lung metastasis or other distant organ metastasis. Differently, more IgA^+^ cells were accumulated in margins or cancer centers of lung metastasis rather than in paracancerous regions of primary cancer of CRC patients. It has been suggested that infiltration of mature plasma cell in tumor stroma is associated with longer survival of CRC patients.^28^ And, IgA positive plasma cells expressing IL-10 and PDL1 is immunosuppressive by inhibiting the CTL effects in oxaliplatin-treated prostate cancer patients.^29^ In this study, one phenomenon we observed is that these IgA^+^ cells accumulated in margins or cancer centers of lung metastasis usually expressed high levels of PD1, which can explain why these cells are not helpful for formation of tertiary lymphoid structures (TLSs) and killing effects of T cells on tumor cells.

We also found that Erbin^+^ B lymphocytes played the key role in lung metastasis of CRC. Erbin, an adaptor for the receptor Erbb2/Her2,^2,30^ plays the key role in cancer progression in many types of cancer including breast cancer, colon cancer, and liver cancer.^31–33^ Interestingly, a recent study reported that Erbin mutation in tumor cells triggered effective immune responses from a colon cancer patient with lung metastasis.^34^ Nevertheless, the role of Erbin in the tumor microenvironment was still unclear. In this study, we found that Erbin was differently expressed in primary cancer and lung metastases of CRC patients. When we further analyzed Erbin expression in stromal cells of CRC, we found a positive correlation between the IgA^+^ and Erbin^+^ stromal cells in IM area of CRC patients. Moreover, using Erbin full knockout and B-cell-specific knockout mouse models of lung metastasis of CRC, we found that Erbin^+^ B cells especially plasma cells (CD138^+^ or IgA^+^ CD138^+^ cells) played the important role in lung metastasis of CRC. Notably, physiologically, the number of B220^−^ CD138^+^ cells and IgA^+^ CD138^+^ cells in lung is about twice that in wild-type mice, suggesting the key role of Erbin in B-cell maturation. As we know, B cells or plasma cells have several critical functions in the antitumor immune response. For example, it can secrete tumor-specific antibodies, or mediate antitumor effects by participating in the presentation of antigens to CD4^+^ and CD8^+^ T cells.^35–39^ In this study, we are trying to explore the mechanisms by which IgA^+^ CD138^+^ cells participate in antitumor immunity. We found that Erbin-regulating B cells could not only inhibit PDL1 expression of tumor cells, but also promote CD8^+^ T cells to kill tumors in vitro. The activated B cells have the ability to kill tumors, but there was no difference in antitumor effect between Erbin-deficient B cells and WT B cells. Only when CD8^+^ T cells exist, Erbin-deficient B cells will cooperate with them to exert a strong ability to kill tumors. The above results suggested that Erbin^+^ lymphocytes were related to the progress of antitumor immunology response mediated by IgA^+^ plasma cells interacting with CD8^+^ T cells.

One of the key mechanisms for the role of Erbin in lung metastasis is that Erbin is required for TGFβ mediated suppression of migration of IgA^+^ CXCR5^+^ cells and Erbin knockout attenuates TGFβ mediated suppression of migration of IgA^+^ CXCR5^+^ cells. In the lung metastases from Erbin^−/−^ mice, the expressions of CXCL13/CXCR5 in B cells were significantly increased compared with that from control WT mice. Importantly, the numbers of IgA^+^ CXCR5^+^ cells in the lung metastases from Erbin^−/−^ mice were about 5–10 times that from WT mice. CXCL13/CXCR5 is very important for activation of B cells, which promote naive B movement to FDC (follicular dendritic cells)^3^ within the follicles. Expression of CXCR5 is maintained when B cells encounter antigens and interact with Tfh cells.^40–42^ In our study, we found that more CXCR5 stimulated by CXCL13 ligand was expressed on IgA^+^ cells in lung metastases from Erbin^−/−^ mice, which led to more IgA^+^ B cells accumulated in lung metastases. In our study, TGFβ, IL-21, IL-6, and IL-4, associated with production of IgA or B-cell differentiation, development or proliferation^43,44^ were differentially expressed in the lung metastases from Erbin^−/−^ and WT mice. Interestingly, we found that TGFβ levels in lung metastases from Erbin^−/−^ mice were greatly decreased compared with that in wild-type mice. We tested the effects of TGFβ or CXCL13 on migration and differentiation of CXCR5^+^ IgA^+^ cells, respectively. CXCL13 made little difference on chemotaxis of CXCR5^+^ IgA^+^ cells from either Erbin knockout or wild-type mice while Erbin knockout greatly attenuated TGFβ mediated suppression of migration of IgA^+^ CXCR5^+^ cells, suggesting the effects of Erbin on chemotaxis of CXCR5^+^ IgA^+^ cells are partly dependent on TGFβ.

Moreover, we found another key mechanism that Erbin knockout inhibited PD1 expression in B cells especially in IgA^+^ cells by STAT6-STAT3 pathway. PD1 is one of the most important immune checkpoints. Some evidences showed that PD1 or PDL1 on B cells can be immunomodulated in some tumors, such as breast cancer, liver cancer, colorectal cancer, and pancreatic cancer.^45^ The use of PD1 or PDL1 antibodies will block the interaction between PDL1 on tumor cells and PD1 on T cells, and restore the antitumor activity of T cells.^46^ PDL1^+^ PC cells have been shown to have immunosuppressive properties in solid tumors in studies of prostate cancer and spontaneous liver cancer. Removal of such cells will enhance the killing capacity of CD8^+^ T cells.^29,47^ In this study, we found that Erbin regulated a class of IgA^+^ CD138^+^ cells highly expressing PD1 and promoted lung metastasis of CRC. Erbin knockout or Erbin knockdown significantly inhibited PD1 expression in B cells in vitro and in vivo. Further evidences showed that Erbin knockout inhibited PD1 expression in B cells especially in IgA^+^ cells by STAT6-STAT3 pathway. In B cells isolated from Erbin^−/−^ mice, we found that the low expression of PD1 was related to upregulation of phosphorylation of STAT6. In B cells isolated from wild-type mice or Erbin^−/−^ mice, the number of B220^+^ PD1^+^ B cells was increased significantly when STAT6 was inhibited by AS1517499 or stably knocked down by shRNA, suggesting STAT6 was required for Erbin-regulating PD1 expression. Further evidences showed that Erbin interacts with STAT6 with the help from STAT3. Erbin promoted the Tyr641 phosphorylation of STAT6 through the STAT3-STAT6 complex and further enhanced the expression of PD1. In the meantime, we found that Erbin was required for IL-6, TGFβ, and IL-21 inducing Tyr641 de-phosphorylation of STAT6 in B cells. It has been reported that STAT6 promotes immunoglobulin class switching to IgE and IgG1 and the expression of molecules on the cell surface responsible for antigen presentation in B cells.^48^ Our study suggested that a key role of Erbin in PD1 expression helps the association of STAT3-STAT6.

Nevertheless, we found that the roles of Erbin in immune cells are very complex. The role of Erbin of immune cells in lung metastasis of CRC needs further confirmation. Interestingly, in orthotopic tumor models of CRC with spontaneous metastasis by trans-anal injection (TRAI) of murine colon cancer cells submucosally into the distal and posterior rectum of mice, we found that there were more lung metastases and liver metastases in the WT mice than that in Erbin knockout mice (data not shown). The role of Erbin in other immune cells, such as T cells, monocytes, and so on is worthy of further exploration. More cKO mice for specific cell types are needed to further study the role of Erbin of immune cells in liver metastasis of CRC. In addition, in the future, targeting Erbin based on the different expressions of Erbin in various immune cells in the tumor microenvironment of CRC patients might provide the potential option for treatment of lung metastasis of CRC.

In summary, our study uncovered a key role of Erbin in regulating IgA^+^ PD1^+^ B cells in lung metastasis of CRC. Targeting Erbin greatly suppressed lung metastasis of CRC by inhibiting PD1 expression of IgA^+^ B cells, promoting aggregation of IgA^+^ B cells, and increasing the killing effects of CD8^+^ T cells on tumor cells.

## Materials and methods

### Animal models

C57BL/6 mice were purchased from Shanghai Slac Laboratory Animal Co. Ltd, and Erbin knockout (Erbin^−/−^) mice, in which exon 16 of *Erbin* gene was deleted by a CRISPR/Pro System, were established in our lab. Erbin^loxp/loxp^ mice kindly gifted by Dr. Tianming Gao from Southern Medical University, were crossed with B6.129P2(C)-Cd19^tm1(cre)Cgn/J^ (CD19-cre), which were purchased from the Jackson Laboratory. Erbin^−/−^ and Erbin^L/L^ mice were crossed to C57BL/6 for at least ten generations and were used for experiments. All mice were maintained under SPF conditions of Soochow University Animal Center. In pulmonary metastasis of colon cancer model, mice at 6–8 weeks of age were injected via tail vein with CMT93 and MC38 cells (1 × 10^6^ per mouse). The resources of mouse CRC cell lines were listed in Table S5, and cultured in DMEM containing 10% fetal bovine serum. Mice were killed on Day 30, when lung and body were weighed and the ratio of the lung weight to the body weight was calculated.

### Cases

The information of patients used for transcriptome sequencing is in the supplementary Table 1. Information of 91 CRC patients without metastasis (cohort1) and 117 CRC patients with metastasis (cohort2) is shown in the supplementary Table 2a, b. Information of CRC patients with lung metastases used for IHC analysis is in Table S3. The cases come from the Department of Pathology, Sun Yat-sen Memorial Hospital, Guangzhou, China.

### B cell isolating and adoptive lymphocyte transfer

B cells were harvested from the bone marrow or spleen of Erbin^−/−^ and cKO mice bearing MC38 cells for 7 days. BD IMag™ Mouse B Lymphocyte Enrichment Set was used to isolate B cells (cells of >90% purity, and >90% viability).

On the 7th day after injecting tumor cells into mice i.v., a total of 1 × 10^6^ sorted B cells in 200 μl PBS were injected i.v. into the recipient mice once only. Three weeks after the adoptive transfer experiments, mice were killed. Lungs were photographed, and the weight of lung and body were recorded. And then, some tissues were fixed for immunohistochemistry or immunofluorescence, and some of them were used to extract lymphocytes.

### Single-cell RNA sequencing

B220^+^ cells from tumors were sorted into Dulbecco’s PBS + FBS (Biological Industries, BI) as aforementioned and retained on ice. Sorted cells were then counted and assessed for the dead cells with Trypan blue by Nikon Eclipse TS100. The cells were then resuspended at 13,105–23,105 cells/mL with a final viability of >90% as determined using the Countess. Single cells were encapsulated into emulsion droplets using Chromium Next GEM Single Cell 5*'* Library and Gel Bead Kit v1.1 (10× GENOMICS). ScRNA-seq libraries were constructed using Chromium Single Cell 5*'* Library Construction Kit (10× GENOMICS) according to the manufacturer’s protocol. Reverse transcription and library preparation were performed on an ABI Veriti96-Well Thermal Cycler. Amplified cDNA was purified using SPRIselect beads (Beckman Coulter) and sheared to 250–400 bp. Qualification was performed using Qubit 3.0 Fluorometer. Libraries were sequenced on an Illumina Nova Seq 6000 system.

### Single-cell RNA-Seq data preprocessing

ScRNA-Seq data was processed using 10× Genomics CellRanger (v3.0.2) pipeline. The resulting fastq files were passed to CellRanger’s count, which aligned all reads against mouse (mm10) genomes using the STAR aligner. The data from libraries were pooled by running through CellRanger aggr to obtain unique molecular identifiers (UMIs) matrices. Both mkfastq and count were run with default parameters.

### Isolation of mouse lymphocytes

Single-cell suspensions were prepared from lung, liver, spleen, intestinal epithelium (including small intestine and colon), blood, and bone marrow.

For lung and liver, mice were euthanized and lung and liver were perfused with cold PBS. Afterward, tissues were dissected into fragments of 1 mm diameter, digested in RPMI-1640 medium containing 1 mg/ml of collagenase type IV enzymatically digested at 37 °C for 30 min in 5 ml of digestion buffer. The digested tissues were then successively passed through 70 µm sterile nylon filter to enrich for leukocytes and washed with 10 ml of serum-free RPMI-1640 medium. Single-cell suspension was transferred to a 15-ml conical tube and centrifuged for 5 min at 1200 rpm, 4 °C, and supernatant was discarded. The cell pellet was resuspended in 5 ml of 40% Percoll, and centrifuged at 2000 rpm for 15 min at 4 °C with free-brake and low-accelerator conditions. Red blood cells were removed by RBC lysis solution. At last, the cell pellet was resuspended in 1 ml PBS containing 2% FBS and count viable nucleated cells in a hemacytometer using trypan blue dye exclusion.

For spleen, peripheral blood and bone marrow, spleens were isolated from mice and mechanically disrupted in RPMI-1640 medium containing 2% FBS using frosted glass slides to generate a single-cell suspension. Then the cells liquid was passed through 70 µm sterile nylon filter to enrich for leukocytes and washed with 10 ml of serum-free RPMI-1640 medium. Single-cell suspension was transferred to a 15-ml conical tube and centrifuged for 5 min at 1200 rpm, 4 °C, and supernatant was discarded. Red blood cells were removed by RBC lysis solution. At last, the cell pellet was resuspended in 1 ml PBS containing 2% FBS and count viable nucleated cells in a hemacytometer using trypan blue dye exclusion.

Peripheral blood was collected by fundus venous plexus with EDTA as anticoagulant, and washed by PBS containing 2% FBS. Then, the steps after transferring from the cell suspension to the 50-ml tube were the same as the extraction of splenic lymphocytes.

Bone marrow was removed from femurs, tibiae, and hips by flushing with RPMI-1640 medium containing 2% FBS. Then, the steps after transferring from the cell suspension to the 50-ml tube were the same as the extraction of splenic lymphocytes.

For small intestine and colon, in brief, Peyer’s patches and fat tissue were carefully removed. The small intestine and colon were opened longitudinally, washed in RPMI-1640 Medium, and cut into 5 mm pieces, which were transferred to a 50 ml conical tube (Falcon 2070) containing 25 ml EDTA-RPMI medium including 10 mM/L HEPES, 25 mM/L NaHCO_3_, 1 mM/L EDTA, 1 mM/L DTT and 2% FBS, and 40 μg/ml penicillin and streptomycin. The tube was shaken at 37 °C for 30 min (horizontal position; orbital shaker at 280 rpm). Cell suspensions were collected in a new 50 ml conical tube and passed through a 70 μm strainer to deplete cell debris and sticky cells (crude cell preparation) and centrifuged at 1200 rpm at 4 °C for 6 min. Cell pellets were resuspended with EDTA-RMPI medium, filtered through 70 μm nylon mesh, and then centrifuged at 1200 rpm at 4 °C for 6 min. Subsequently, the pelleted cells were suspended in 40% Percoll. Percoll was diluted with PBS to make 1x Percoll. Cells were suspended with 16 ml of 40% Percoll in 15-ml tubes. Two milliliters of 75% Percoll was added into the lower layer of 40% Percoll in each 15mltube. IELs were collected by harvesting the center layer between 40 and 70% Percoll after being centrifuged at 2000 rpm for 15 min at 20 °C with free-brake and low-accelerator conditions.

### Flow cytometry

Aliquots of 1 × 10^6^ cells above tissues were stained with fluorochrome-conjugated antibodies (listed in Table S4). Flow cytometric acquisition was performed using FACScan (BD Biosciences), and data were analyzed using FlowJo software.

For intracellular cytokine Perforin, GrzB, and IFNγ production by CD8^+^ and CD4^+^ cells staining, cells were restimulated for 1 h with 0.5 ng/ml PMA and 0.5 ng/ml ionomycin in RPMI-1640 medium containing 10% FBS to allow intracellular cytokine accumulation. Then, Protein Transport Inhibitor was added to the cells and incubated for 3–5 h with intermittent mixing. For the cytokine stains, fixed and permeabilized with Fixation/Permeabilization solution, and stained in PBS containing 2% FBS.

For Foxp3 and Ki67 staining, we followed the procedure of the staining procedure of the eBioscience Transcription Factor staining buffer set.

For p-STAT6 staining, cells were fixed by Fixation Buffer and permeabilized by Perm Buffer III, and then stained with fluorochrome-labeled p-STAT6 in Stain Buffer (FBS).

### Cellular identification and clustering

For each sample, the gene-barcode matrix was passed through the R (v3.5.3) software package Seurat (v2.2.1)^47^ (https://satijalab.org/seurat) for all downstream analyses. To remove doublets and poor-quality cells, cells were excluded from subsequent analysis if they were outliers in terms of number of the genes, UMIs, and the percentage of mitochondrial genes using Seurat. T-distributed stochastic neighbor embedding (t-SNE) and modularity heatmap were created for graph-based cluster identification and subsequent dimensionality reduction. Marker genes for the individual cluster were identified using Seurat’s FindAllMarkers and only significantly upregulated markers (minimum log.FC ≥ and FDR ≤ 1 × 10^−75^) were represented.

### Plasma cell induction

To induction plasma cell, B cells isolated from mice were incubated in RPMI-1640 medium containing 10% FBS and 1 μg/mL CD40L plus 50 ng/mL recombinant IL-21. Culture supernatants were collected on the 6th day for detection of immunoglobulins.

### CD8^+^ cell isolating and activated

Mouse CD8^+^ T Lymphocyte Enrichment Set-DM was used for isolating CD8^+^ T cells from spleen of mice. Anti-CD3, anti-CD28, and IL-2 were used to activate CD8^+^ cells in vitro.

### Co-culture experiments

In order to analyze the effect of B cells on T cells and tumor cells, and the effect of T cells on the IgA^+^ in B cells, we used the following co-culture system in vitro.

B cells (4 × 10^6^ per well) from WT or cKO mice were co-culture with MC38 cells (3.5 × 10^5^ per well), while the medium contained 10 μg/ml LPS was added in medium to activate B cells. In some cases, the MC38 cells were developed for expressing FITC stably. After 24 h, CD8^+^ T cells (3 × 10^5^ per well) isolated from WT mice were added to the cultures with or without B cells for 48 h. The proportion of IgA^+^ PD1^+^ cells, and MC38 cells expressing PDL1 were analyzed by flow cytometry. CTL assay was used to detect the killing ability of B cells, CD8^+^ T cells, or B cells combined with CD8^+^ T cells on tumor cells.

To analyze whether cytokines regulated IgA^+^ B cells or PD1^+^ B cells by Erbin, 50 ng/ml IL-4, 100 ng/ml IL-6, 50 ng/ml IL-21, 5 ng/ml TGFβ (listed in Table S6) were added in B cells isolated from spleen of WT and cKO or Erbin^−/−^ mice. The expression of PD1, p-STAT6, p-STAT3, STAT6, and STAT3 was detected by western blotting.

### Inhibition of STAT6 in vitro

100 nM AS1517499 was added in B cells (2 × 10^7^ cells) sorted in vitro for 48 h. Meanwhile, the lentivirus inducing knockdown of STAT6 (shSTAT6) was transfected into 293T cells.

### In vitro B-cell migration assay

Migration assays were performed in a Transwell system with 5-mm pore size (Costar, Cambridge, MA). B cells (5 × 10^5^) were added to the upper chamber, and 10 ng/mL of CXCR5 ligand CXCL13 was added to the lower chamber. After 8 h, the B cells in the upper and lower chambers were harvested and counted separately. The ratios of IgA^+^ CXCR5^+^ B cells in the different chambers were determined by FACS. The ratios of migrated IgA^+^ CXCR5^+^ B cells to total in IgA^+^ CXCR5^+^ B cells were calculated.

In parallel, 5 ng/ml TGFβ was added to the lower chamber for 8 h, and meanwhile, the B cells were left untreated as control.

### CTL assay

Cytotoxic activity was measured by LDH release from MC38 cells using the CytoTox 96® Non-Radioactive Cytotoxicity Assay kit.

### ELISA

Peripheral blood of mice and culture supernatants from vitro co-culture experiment or induction of plasma cells were analyzed by IgA, IgG, and IgM ELISA kits. All experiments were performed according to the manufacturer’s instructions. Each serum was tested in duplicate. Results are expressed as OD at 450 nm.

### Histology

Lungs were fixed in 4% paraformaldehyde, embedded in paraffin, sectioned, stained with haematoxylin and eosin, Sirius Red, and processed for immunohistochemistry. The number of tumors was counted on the largest section of the lungs.

### Immunohistochemistry

The tissue specimens were fixed in 10% formalin, embedded in paraffin, and sectioned. After dewaxing and rehydration, the specimens were incubated in citrate buffer at 100 °C for 10 min for antigen retrieval and incubated blocking buffer (5% normal goat serum in PBS) for 1 h at RT. Then, tissue sections were incubated overnight at 4 °C with the primary antibodies (listed in Table S4). After washing three times, tissue sections were incubated with biotinylated secondary antibody reagents for 20 min at room temperature. DAB solution was added after washing with PBS. Then, the slides were counter stained with haematoxylin.

For evaluation of immunohistochemical variables, experiment was performed by two independent observers who were blinded to the clinical outcome. At low power, the tissue sections were screened, and the five most representative fields were selected. Thereafter, to evaluate the density of these genes, the respective areas of intra-tumoral region and paracancerous region were measured at ×400 magnification, and the nucleated IgA^+^, CD138^+^, PD1^+^, CD8^+^, and Erbin^+^ stroma cells in each area were counted.

### Immunofluorescence

Tissues for immunofluorescence were fixed with 4% paraformaldehyde for 15 min at room temperature, washed with PBS, and permeabilized with 0.2% Triton X-100 in PBS for 15 min. Thereafter, tissues were blocked in PBS with 2% BSA for 1 h at room temperature. After blocking, samples were incubated with primary antibodies (listed in Table S4) specific for anti-IgA and anti-CXCR5 overnight at 4 °C. Secondary antibodies were incubated for 1 h at room temperature. DAPI was then used for counterstaining the nuclei and images were obtained by laser scanning confocal microscopy (OLYMPUS FV3000). To evaluate the density of IgA^+^ CXCR5^+^, regions were measured at ×648 magnification, and the nucleated cells in each area were counted using OLYMPUS FV31S-SW Image Analysis Software.

### Western blotting

Cells were collected in cell lysis buffer containing protease and phosphatase inhibitor, and lysates were sonicated and centrifuged at 4 °C. Per lane, whole-cell lysate was separated on 8 or 10% SDS-acrylamide gels and transferred on Immobilon PVDF membranes (Millipore). The membranes were probed with primary antibodies (listed in Table S4) overnight at 4 °C and incubated for 1 h with secondary peroxidase-conjugated antibodies. Chemiluminescent signals were then detected by ECL system.

### Co-immunoprecipitation (Co-IP) assay

Myc-Erbin, flag-STAT3, flag-STAT6, and empty vector plasmids were transfected alone or together in vitro to further detect the interaction of Erbin and STAT6 in 293T cells, which were collected in cell lysis buffer.

Meanwhile, B cells from spleen of mice were collected in cell lysis buffer to detect the interaction of Erbin and STAT6.

Furthermore, lymphocytes were separated from spleens of Erbin^−/−^ and WT mice. After LPS activation in vitro, cytokines IL-6, TGFβ, and IL-21 were added, respectively. By observing the protein amount of Erbin/STAT6 pulled by STAT3, we determined which single factor mainly affects the Erbin-STAT3-STAT6 complex in lymphocytes.

293T cells, B cells, or lymphocytes from spleen of mice were collected in cell lysis buffer. Supernatant was incubated with appropriate antibody (listed in Table S4) for at least 90 min at 4 °C followed by incubation overnight with Protein G agarose. After overnight incubation, the agarose beads were washed four times with cold lysis buffer, incubated for 10 min at 108 °C with loading buffer, and subjected to SDS-PAGE and western blotting analysis.

### RNA isolation and qRT-PCR

RNA from tissue was isolated using the Trizol according to the manufacturer’s instructions. RNA from lymphocytes was isolated by RNeasy Micro Kit. For mRNA, cDNA was generated from 1 μg total RNA per sample using the Transcriptor First Strand cDNA Synthesis Kit. qPCR was performed by using the ABI StepOne Plus and the SYBR® Select Master Mix. mRNA expression was normalized using detection of 18 S Ribosomal RNA. Results are represented as fold induction using the ΔΔCt method with the control set to 1. The sequences of qPCR primers were listed in supplementary Table 7.

### In vivo depletion or inhibition studies

For B-cell depletion, 300 mg per mouse anti-CD19 and 300 mg per mouse anti-B220 were injected i.p. three weeks before tumor inoculation and every 5 days thereafter. B-cell depletion experiments were repeated independently three times with 3–5 mice per group.

For in vivo immune checkpoint PD1 blockade experiments, anti-PD1 antibodies were given i.p. at a dose of 200 mg per mouse 5 days after tumor implantations, and then were given every 5 days.

For siRNA treatment analysis, depletion of Erbin was achieved by i.v. injection of 50 μg 5′-Chol and 2′OMe modified Erbin-siRNA (RiboBio) dissolved in diluted water per mouse 3 days after tumor inoculation, and twice a week thereafter.

After 28 days, the mice were sacrificed to observe the lung metastasis. Some wax blocks were prepared and some were analyzed by flow cytometry. On the 60th day, another batch of mice was sacrificed and survival curves were drawn.

### Statistical analyses

The results are presented as means ± standard deviation (s.d.), and analyses were performed with SPSS version 17.0. Differences between the groups were evaluated by one-way analysis of variance (ANOVA) and correlation analysis was performed using Spearman’s rank correlation. A *P*-value of <0.05 was considered significant.

## Supplementary information

Supplementary Materials

## Data Availability

All data that support the findings of this study are available from the corresponding author upon reasonable request.
