# Supplementary material for: Targeting Erbin in B cells for therapy of lung metastasis of colorectal cancer
Source: Signal Transduct Target Ther. 2021 Mar 12;6:115. doi: 10.1038/s41392-021-00501-x (PMC7952714; doi:10.1038/s41392-021-00501-x)
Supplement: Supplementary file 1 — Supplementary Materials [file 41392_2021_501_MOESM1_ESM.docx]

Supplementary Materials for

**Targeting Erbin in B cells for therapy of lung metastasis of colorectal cancer**

Tong Shen^1#^, Jing-Ling Liu^1#^, Chu-Yi Wang^1#^, Youlutuziayi Rixiati^1^, Shi Li^1, 2^, Ling-Dong Cai^1^, Yuan-Yuan Zhao^1^, and Jian-Ming Li^1, 2*^

Correspondence to: [jianmingli@suda.edu.cn](mailto:jianmingli@suda.edu.cn)

**This PDF file includes:**

Figures. S1 to S5

Tables S1 to S7


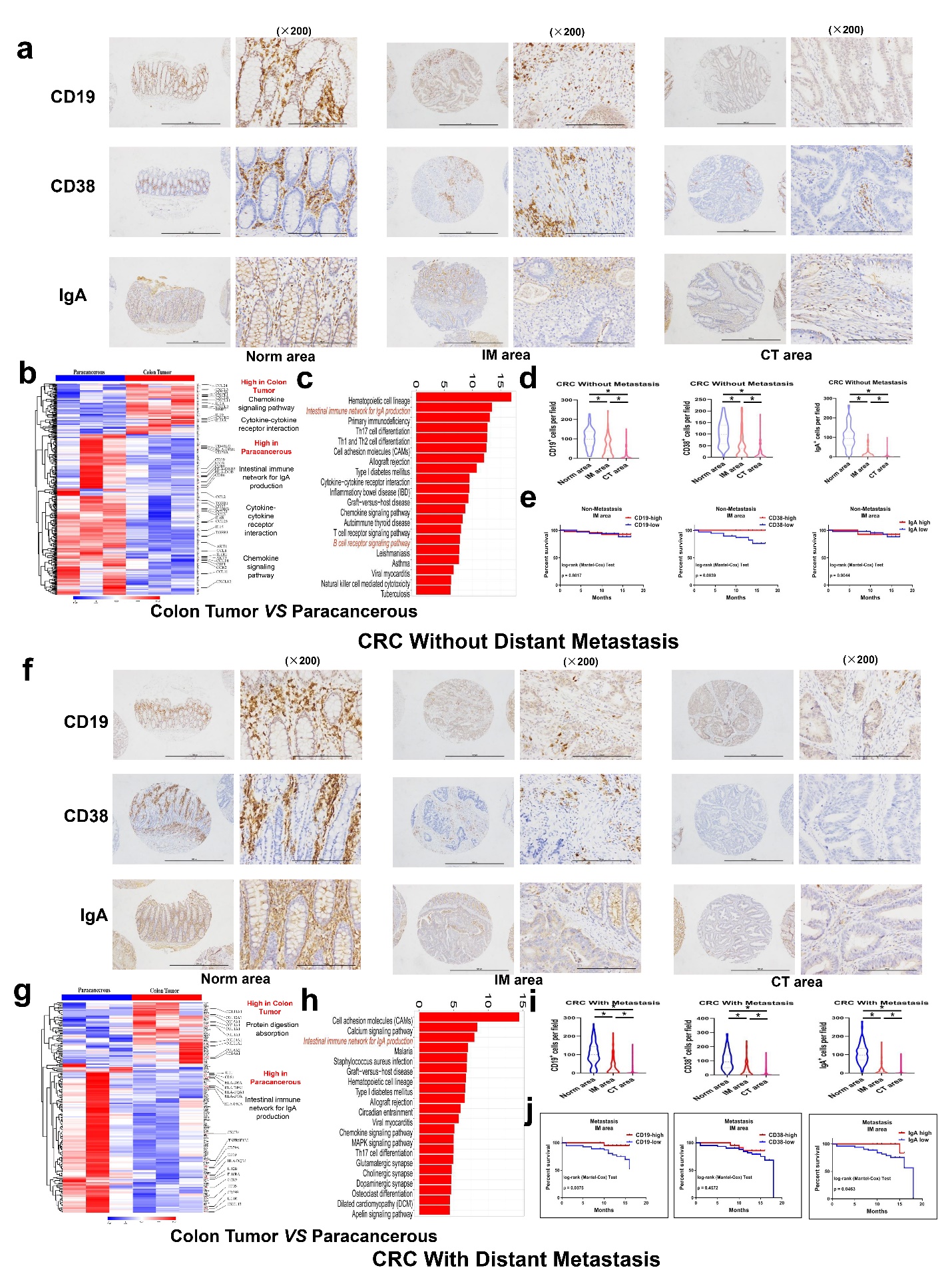


**Figure. S1. Transcriptome analysis and B cells distributions of tumors and adjacent tissues from CRC cancer patients with or without distant metastasis. a,** Immunohistochemical staining of CD19^+^, CD38^+^, and IgA^+^ cells in the adjacent stromal, paracancerous or normal tissue (Norm), invasion margin (IM) and cancer center (CT) areas in the colon tissue of cohort 1 CRC patients (91 patients without distant metastasis). **b-c,** Heatmap representing differential expressed genes (**b**) and KEGG pathway enrichment analyses of differential pathway (**c**) of CRC without distant metastasis between colon tumors and paracancerous tissues. **d,** The number of CD19^+^ cells, CD38^+^ cells, and IgA^+^ cells per field in CRC patients from cohort 1were quantified (×400). **e,** Survival curves of patients in cohort 1 with low and high infiltration numbers of CD19^+^ cells, CD38^+^ cells, and IgA^+^ cells in the IM area. **f,** Immunohistochemical staining of CD19^+^, CD38^+^, and IgA^+^ cells in Norm area, IM area, and CT area in the colon tissue of cohort 2 CRC patients (117 patients with distant metastasis). **g-h,** Heatmap representing differential expressed genes (**g**) and KEGG pathway enrichment analyses of differential pathway (**h**) of CRC with distant metastasis between colon tumors and paracancerous tissues. **i,** Quantification of the number of CD19^+^ cells, CD38^+^ cells, and IgA^+^ cells per field (×400) on cohort 2. **j,** Survival curves of cohort 2 CRC patients with low and high infiltration numbers of CD19^+^ cells, CD38^+^ cells, and IgA^+^ cells in the IM area. **p*<0.05 (Student t test).


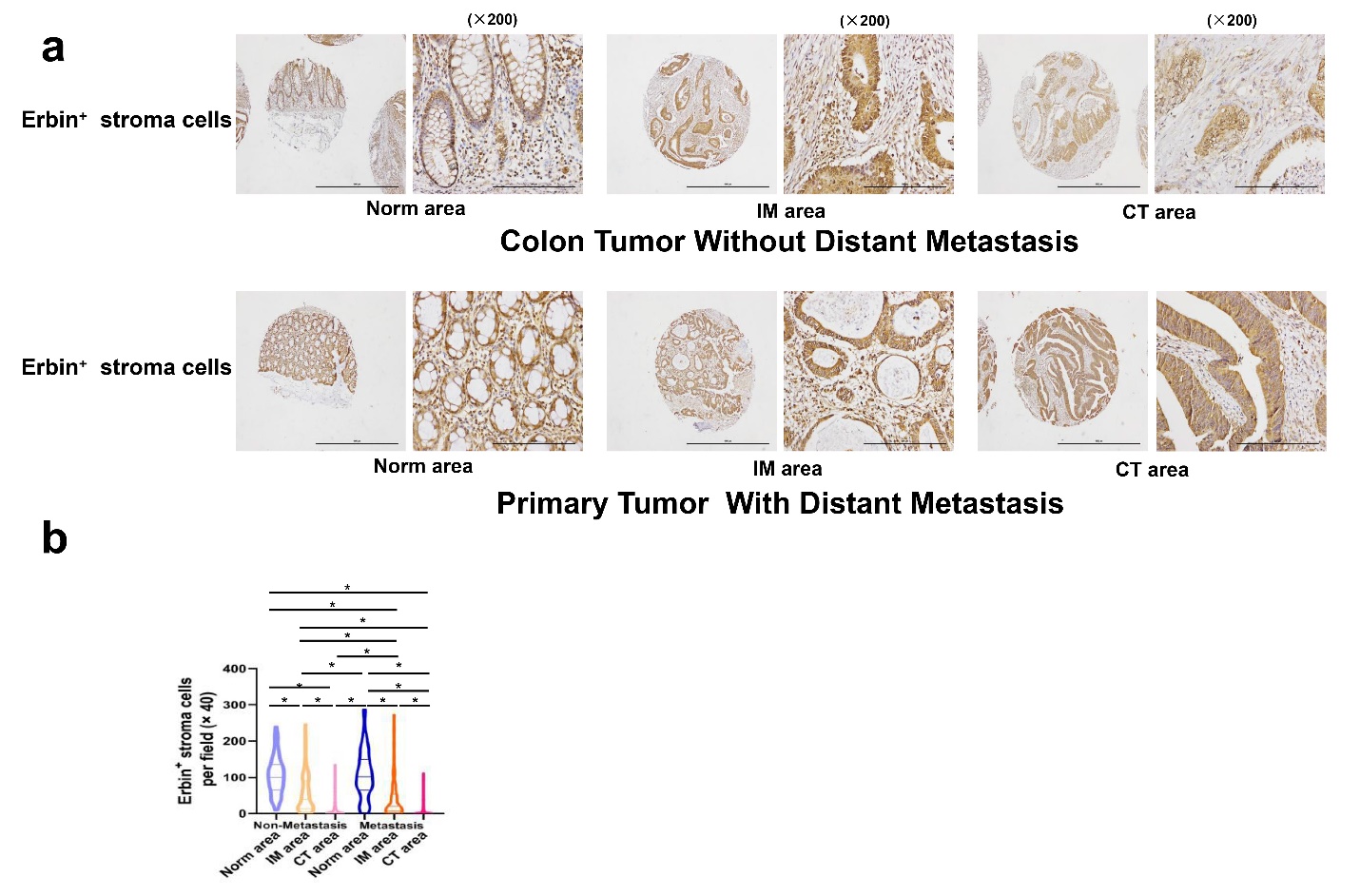


**Figure. S2.** **Erbin^+^ stroma cell distributions of tumors and adjacent tissues from CRC cancer patients with or without distant metastasis. a,** Immunohistochemical staining of Erbin^+^ stromal cells in Norm area, IM area, and CT area of primary tumors from CRC patients (cohort 1 and 2). **b,** Semi-quantification of the number of Erbin^+^ stroma cells mostly lymphocytes per field (×400).


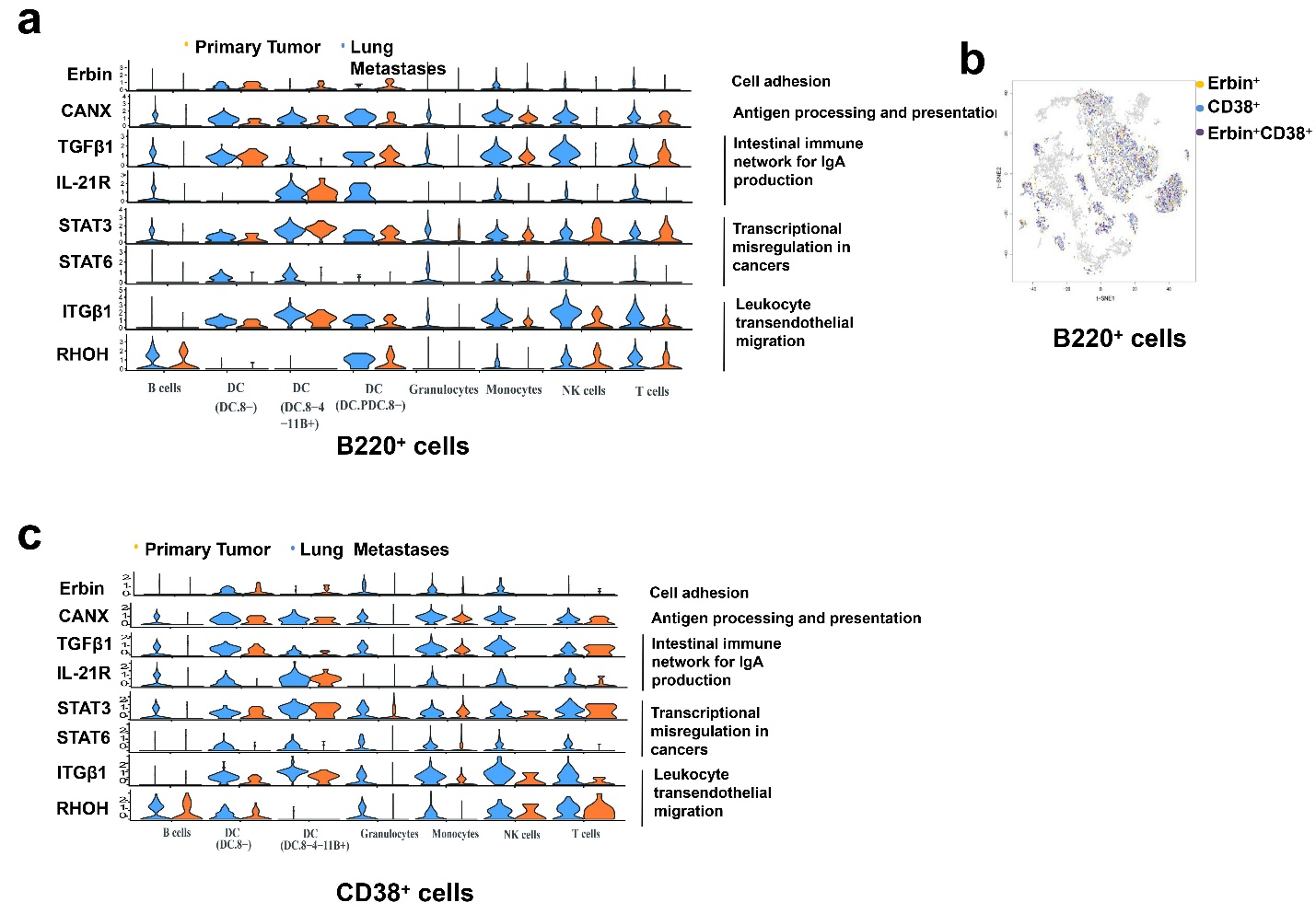


**Figure. S3. Proportion distribution of cells expressing key genes enriched. a,** The violin diagram for the proportion distribution of B220^+^ cells expressing key genes enriched from primary tumor and lung metastases of mouse CRC model by scRNA-seq. **b,** Distribution of Erbin^+^ cells, CD38^+^ cells, and Erbin^+^ CD38^+^ cells isolated from primary tumors and lung metastases of mouse CRC model by scRNA-seq. Graph-based clustering of B220^+^ cells by t-SNE. **c,** The violin diagram for the proportion distribution of CD38^+^ cells expressing key genes enriched from primary tumor and lung metastases of mouse CRC model by scRNA-seq.


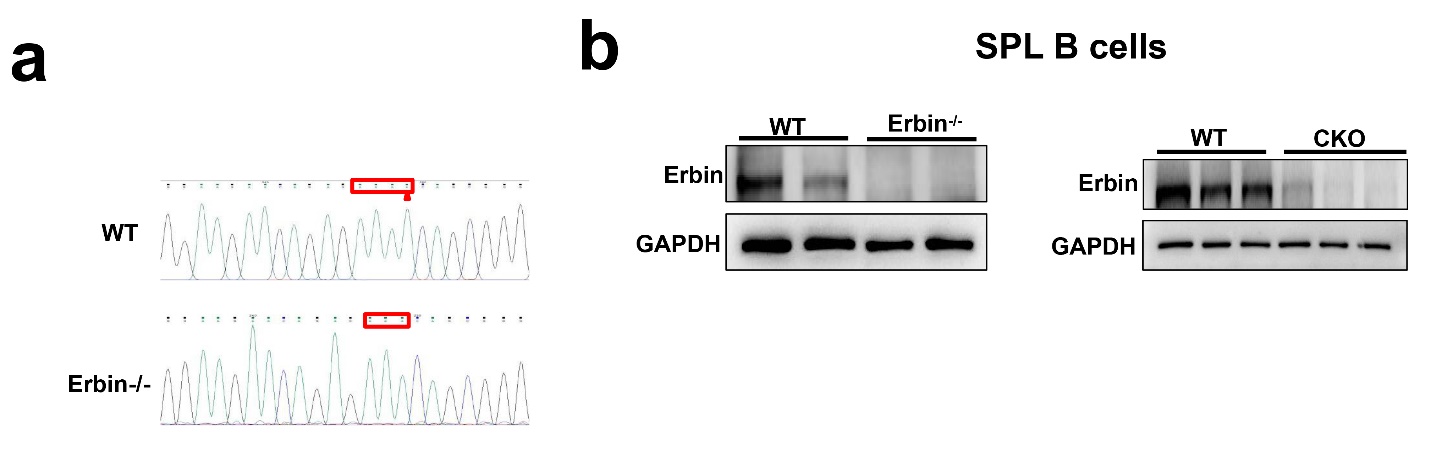


**Figure. S4. Identification of transgenic mice. a,** Identification of Erbin^-/-^ mice by sequencing. **b,** Identification of WT and cKO mice by Western Blotting.


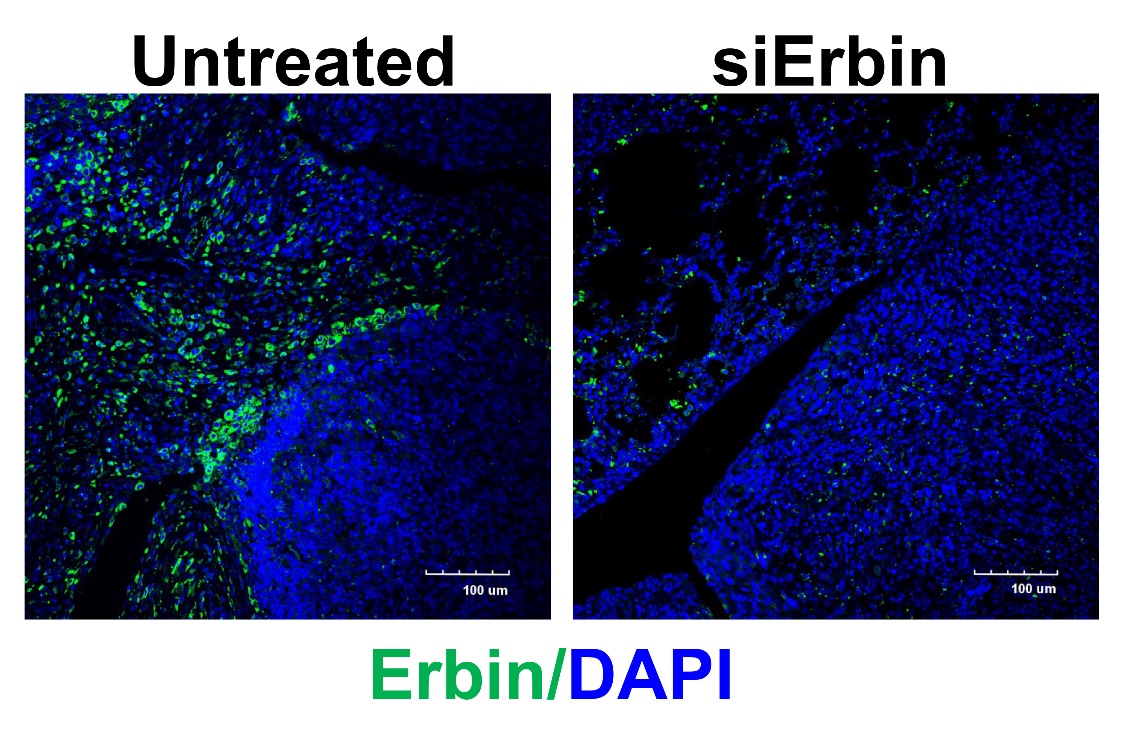


**Figure. S5. Identification of the effect of siRNA targets of inhibiting Erbin expression in mice.** Immunofluorescence of Erbin^+^ cells of lung metastases of untreated and siErbin treating mice, scale bars, 20 μm.

Table S1. Information of CRC patients for transcriptome sequencing





Table S2. Information of CRC patients in cohort 1 and cohort 2

**a,** CRC Patients without metastasis in Cohort1. **b,** CRC Patients with metastasis in Cohort 2.















**Table S3. Information of CRC patients by IHC**

**

**

**Table S4. Antibodies used for flow cytometry, immunohistochemistry, Western Blotting, immunofluorescence and CO-IP.**

**
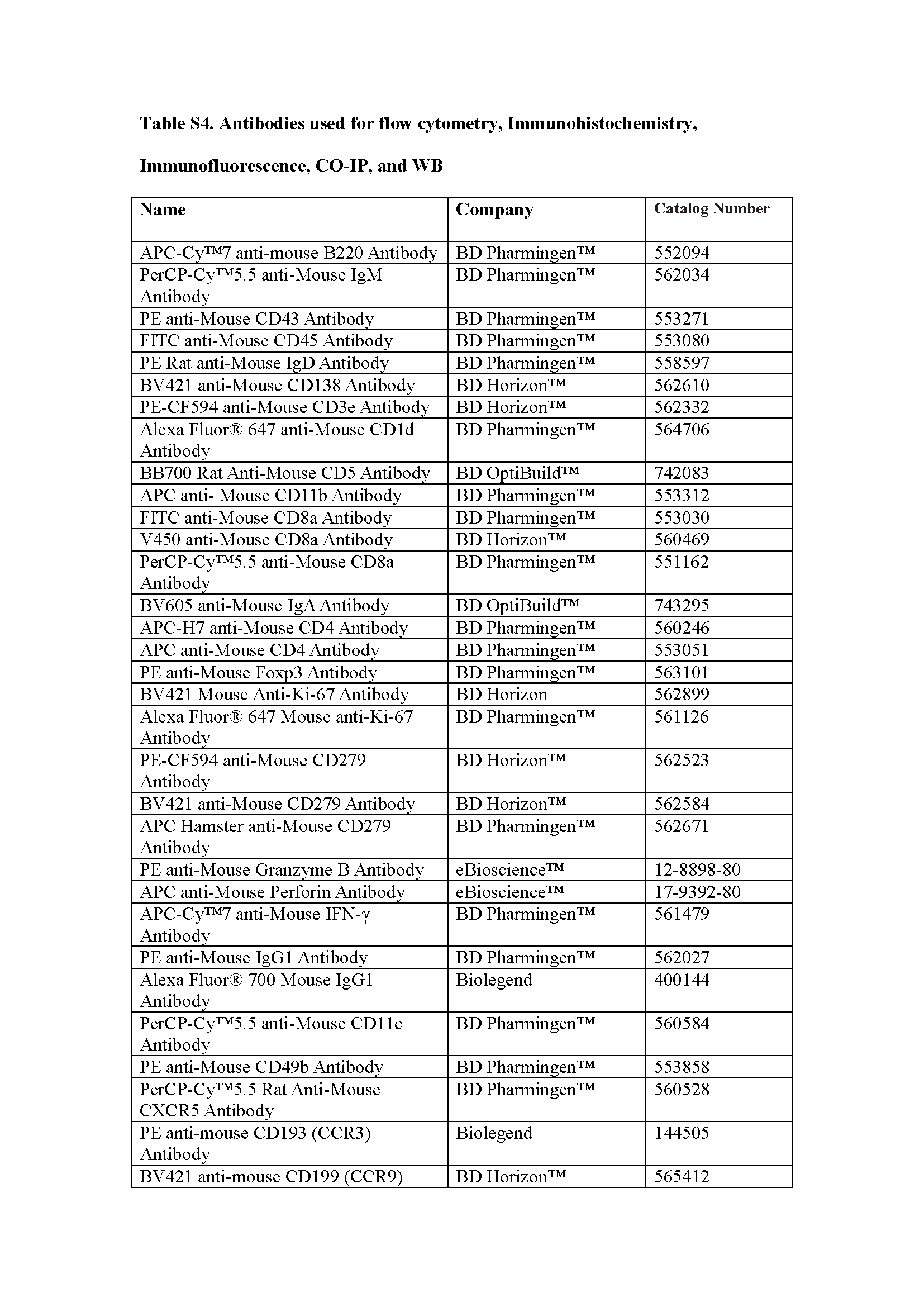
**





**Table S5. Experimental models: organisms/strains**

**
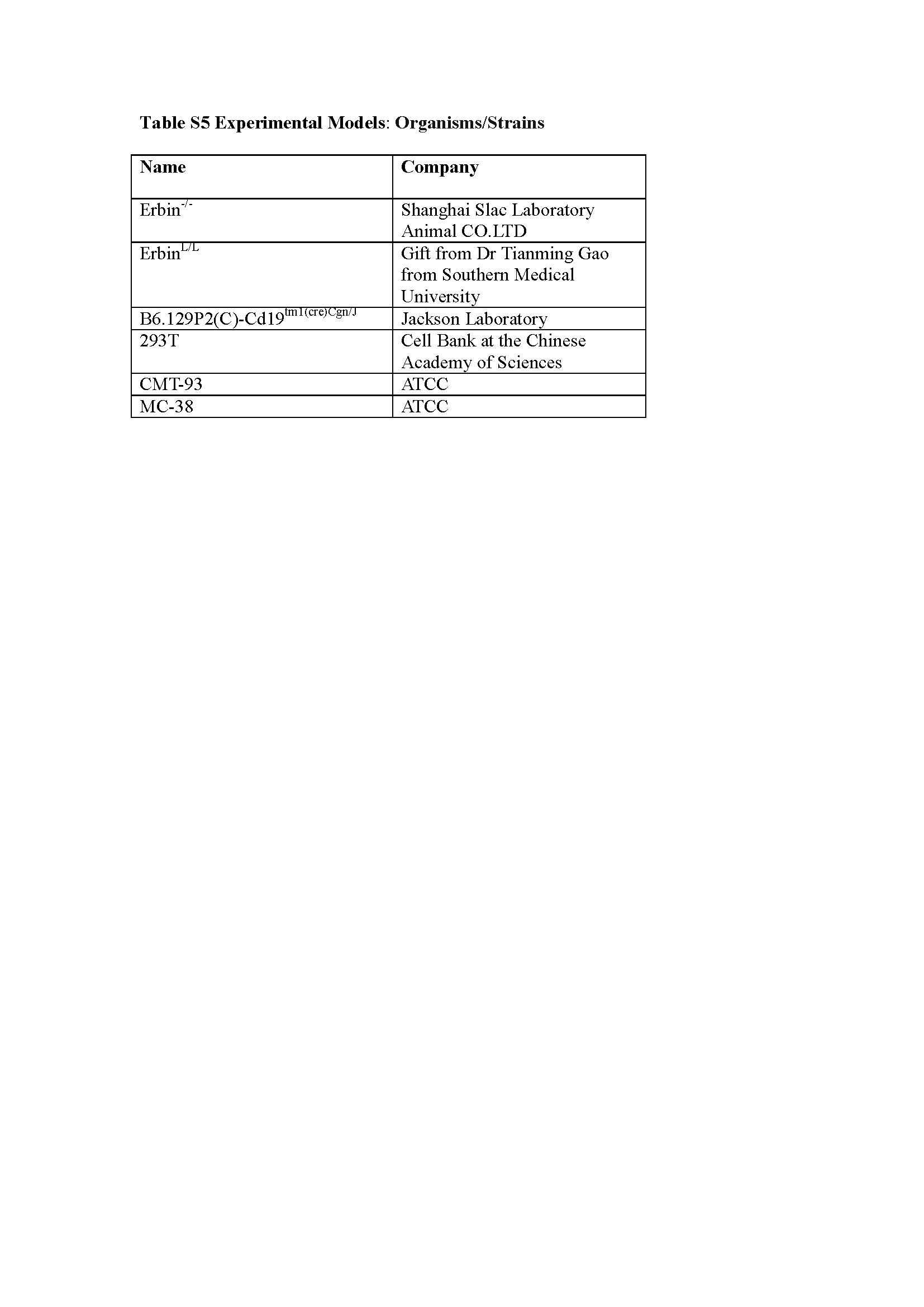
**

**Table S6. Chemicals, peptides, recombinant proteins and critical commercial assays**

**
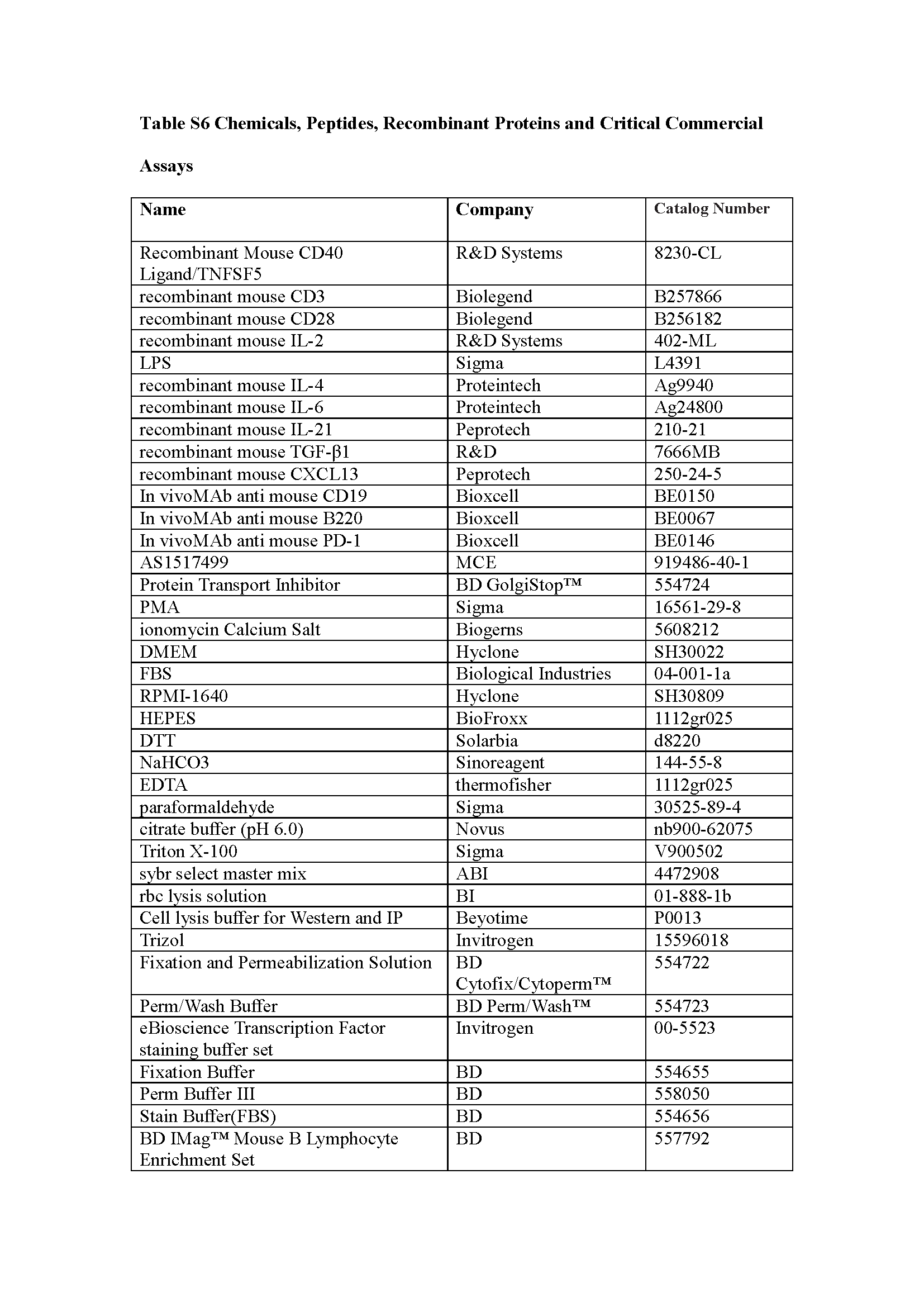
**

**

**

**Table S7. Primers for qRT-PCR**

**
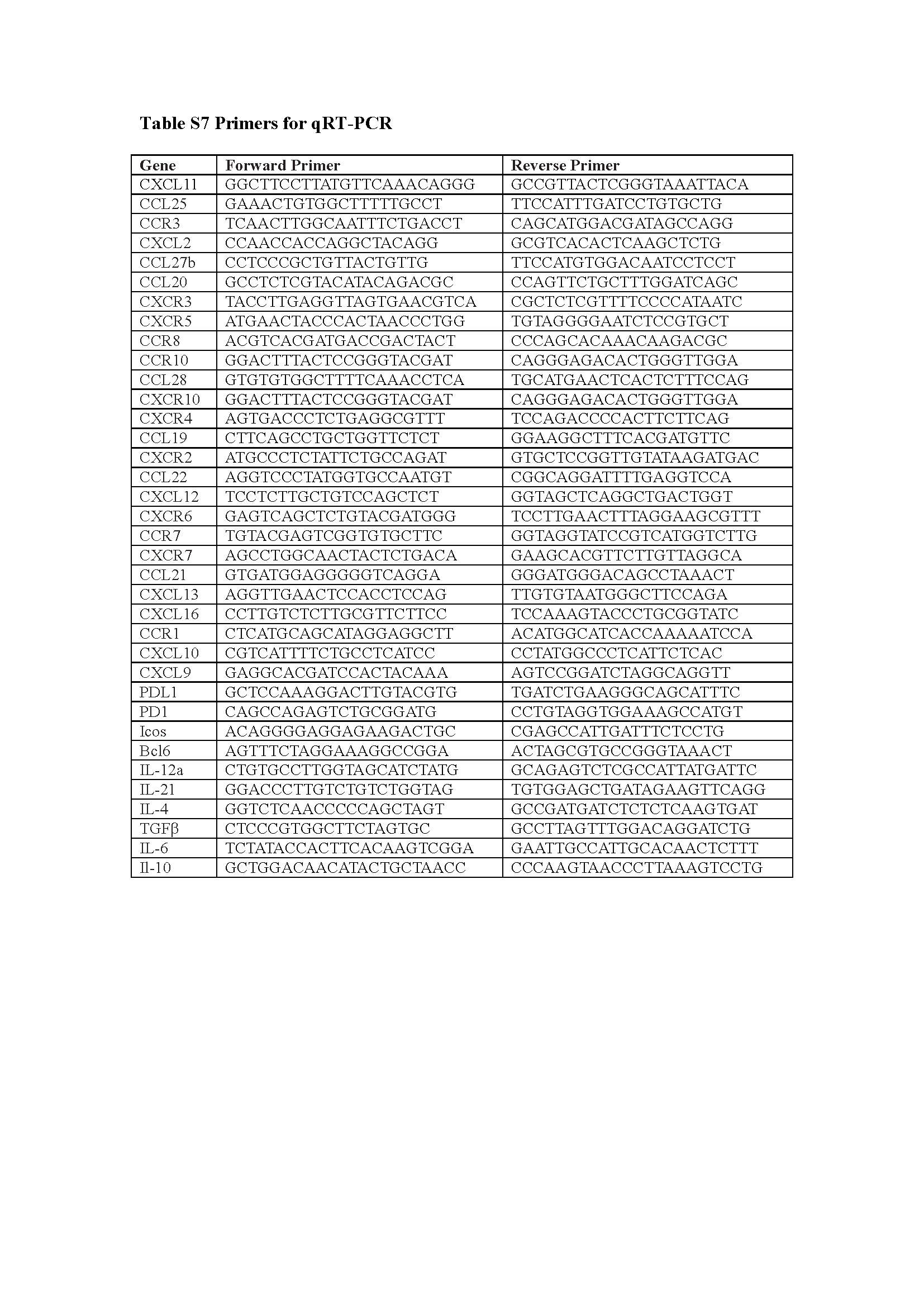
**
